# Isolation and Structure Identification of Novel Brominated Diketopiperazines from *Nocardia ignorata*—A Lichen-Associated Actinobacterium

**DOI:** 10.3390/molecules22030371

**Published:** 2017-02-28

**Authors:** Alba Noël, Solenn Ferron, Isabelle Rouaud, Nicolas Gouault, Jean-Pierre Hurvois, Sophie Tomasi

**Affiliations:** CORINT, UMR CNRS ISCR 6226, UFR Sciences Pharmaceutiques et Biologiques, Université Bretagne Loire, 2 Av. du Professeur Léon Bernard, 35043 Rennes, France; alba.noel@univ-rennes1.fr (A.N.); solenn.ferron@univ-rennes1.fr (S.F.); isabelle.rouaud@univ-rennes1.fr (I.R.); nicolas.gouault@univ-rennes1.fr (N.G.); jean-pierre.hurvois@univ-rennes1.fr (J.-P.H.)

**Keywords:** diketopiperazines, *Nocardia*, lichen-associated actinobacterium

## Abstract

Actinobacteria are well known for their potential in biotechnology and their production of metabolites of interest. Lichens are a promising source of new bacterial strains, especially Actinobacteria, which afford a broad chemical diversity. In this context, the culture medium of the actinobacterium *Nocardia ignorata,* isolated from the terrestrial lichen *Collema auriforme*, was studied. The strain was cultivated in a BioFlo 115 bioreactor, and the culture medium was extracted using an XAD7HP resin. Five known diketopiperazines: cyclo (l-Pro-l-OMet) (**1**), cyclo (l-Pro-l-Tyr) (**2**), cyclo (d-Pro-l-Tyr) (**3**), cyclo (l-Pro-l-Val) (**4**), cyclo (l-Pro-l-Leu) (**5**), and one auxin derivative: indole-carboxaldehyde (**8**) were isolated, along with two new brominated diketopiperazines: cyclo (d-Pro-l-Br-Tyr) (**6**) and cyclo (l-Pro-l-Br-Tyr) (**7**). Structure elucidation was performed using HRMS and 1D and 2D NMR analysis, and the synthesis of compounds **6** and **7** was carried out in order to confirm their structure.

## 1. Introduction

In the field of research on new active compounds from nature, the exploration of original sources of natural products has become more attractive. In this context, the investigation on lichens is an interesting challenge. The symbiotic feature of this organism classically described between a green algae (or cyanobacteria) and a fungus led to the production of interesting metabolites [1], but it is also a long-lasting environment which can host various microorganisms [2,3,4,5]. A plethora of bacteria have been listed from this ecological niche using culture-independent (reviewed by Suzuki et al. [6]) or culture-dependent approaches [2,7]. The three main bacterial phyla found associated with lichens are *Proteobacteria*, *Firmicutes*, and *Actinobacteria* [2,5,8]. The chemistry of some of these bacteria have been already studied, highlighting their ability to produce active compounds [6,9].

**Figure 1:**
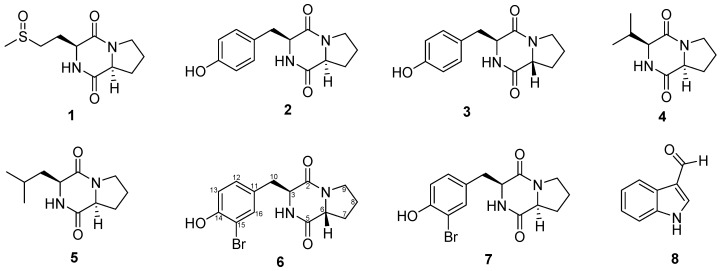
Structure of compounds **1**–**8** isolated from *Nocardia ignorata.*

In our ongoing research to study lichen-associated bacteria, we focused this study on an actinobacterium, relative to *Nocardia ignorata* (16s RNA sequence similarity of 98.58%), previously isolated from a terrestrial lichen *Collema auriforme* [2]. Actinobacteria are well-known for their biotechnological properties, and especially for the production of compounds of therapeutic interest, such as antibiotics (e.g., streptomycin) or anticancer agents (e.g., mithramycin) [10,11]. The chemistry of *Nocardia ignorata* was not yet described, and a preliminary assay on different cell lines revealed an interesting cytotoxic activity of its extract against B16 (murine melanoma) cell lines (IC_50_ 23 ± 3 µg/mL). In the present study, we have isolated two new brominated diketopiperazines and six known compounds from our strain (Figure 1). The aims of this study were to characterize these compounds and to evaluate their cytotoxicity against HaCaT and B16 cell lines.

## 2. Results and Discussion

### 2.1. Chemical Profiling of Extracts and Purification

Two different extracts were obtained from the culture of *Nocardia ignorata*. The first steps of the process are to adsorb metabolites from the medium culture on Amberlite XAD7HP resin. Then, these compounds were eluted from the resin using a mixture of MeOH/Acetone (1/1) leading to a resin extract (RE). After depletion by the resin, the medium was extracted with EtOAc in order to collect compounds not adsorbed on resin affording a supernatant extract (SE).

**Figure 2:**
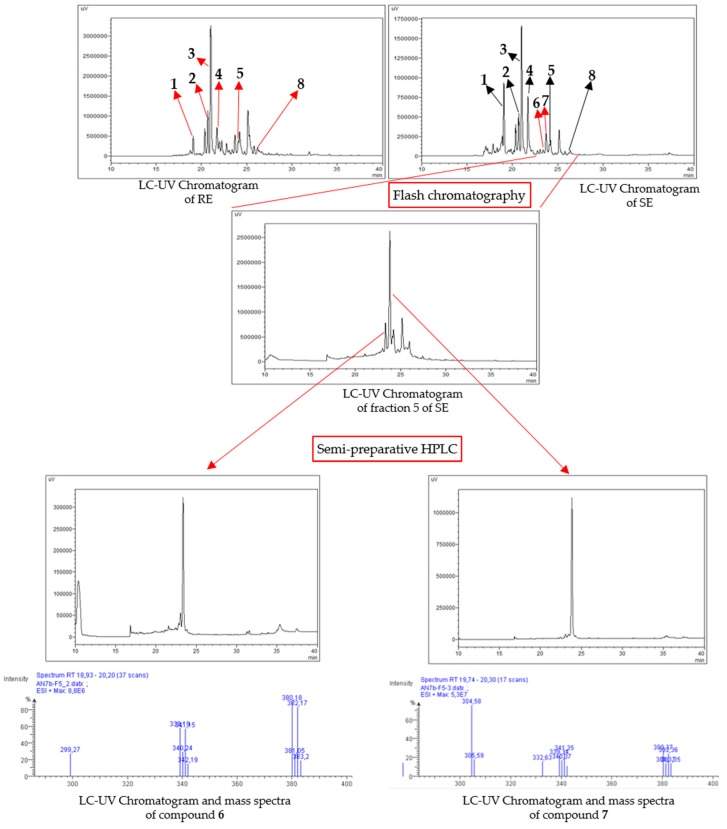
Chemical profiling and isolation process of compounds **6** and **7**. All samples were analyzed at 220 nm on Prevail reversed phase C_18_ column with a gradient of H_2_O (A)/acetonitrile (B) (10 min 100% of A, 30 min from 0% of B to 100% of B, 10 min 100% of B). Supernatant extract (SE) was fractionated using flash chromatography with a reversed phase to afford 14 fractions. Compounds **6** and **7** were isolated from the fifth fraction using semi-preparative HPLC on Prevail C_18_ column. RE: resin extract.

As shown in Figure 2, these extracts were analyzed using HPLC-UV, highlighting that chemical profiles between RE and SE are different. While more polar compounds are present in SE (from 16.5 min to 20.0 min), RE contained less polar metabolites (from 21.8 to 35.0 min). RE and SE extracts were then fractionated with flash chromatography and semi-preparative HPLC to isolate pure compounds.

Compounds **1** (6.9 mg), **2** (5.7 mg), **3** (8.5 mg), **4** (1.1 mg), **5** (4.3 mg), and **8** (5.0 mg) were isolated from the resin extract. This extract was divided into 23 fractions by flash chromatography on normal phase using a gradient of cyclohexane/CH_2_Cl_2_/EtOAc/MeOH. Compounds **1**–**5** and **8** were obtained after purification of fractions 6 (compound **8**), 8 (compounds **4** and **5**), 9 (compounds **2** and **3**), and 21 (compound **1**) using semi-preparative HPLC on a C_18_ Prevail column with a gradient of H_2_O and acetonitrile.

Compounds **6** (1.4 mg) and **7** (2.1 mg) were isolated from the supernatant extract, which was subjected to flash chromatography on reversed phase to afford 14 fractions. The fifth fraction led to compounds **6** and **7** after purification by semi-preparative HPLC using a gradient of H_2_O/acetonitrile.

The culture of *Nocardia ignorata* was stopped after 4 days of growth during the death phase of the bacterial growth. These two new compounds were only produced during this phase.

Six known compounds were isolated from culture media of *Nocardia ignorata.* Five of them are diketopiperazines, including: cyclo (l-Pro-l-OMet) (**1**) [12], cyclo (l-Pro-l-Tyr) (**2**) [13,14], cyclo (d-Pro-l-Tyr) (**3**) [13], cyclo (l-Pro-l-Val) (**4**) [14], cyclo (l-Pro-l-Leu) (**5**) [14,15] and the last one was determined as an auxin derivative: indole-carboxaldehyde (**8**) [15]. The structure of these compounds was elucidated using ^1^H- and ^13^C-NMR data from literature.

### 2.2. Structural Determination of Diketopiperazines ***6*** and ***7***

#### 2.2.1. Structural Analysis

Two new diketopiperazines were identified as cyclo (d-Pro-l-Br-Tyr) (**6**) and cyclo (l-Pro-l-Br-Tyr) (**7**). Compound **7** was isolated as a white amorphous powder from the supernatant extract. Analysis by HR-ESIMS (High Resolution ElectroSpray Ionisation Mass Spectrometry) revealed an isotope pattern of a monobrominated compound with a molecular formula of C_14_H_15_BrN_2_O_3_ (*m*/*z* 339.0329 [M + H]^+^, calcd. 339.0339). The ^1^H-NMR spectrum showed similar signals with those of cyclo (l-Pro-l-Tyr) excepted for the aromatic protons.

**Figure 3:**
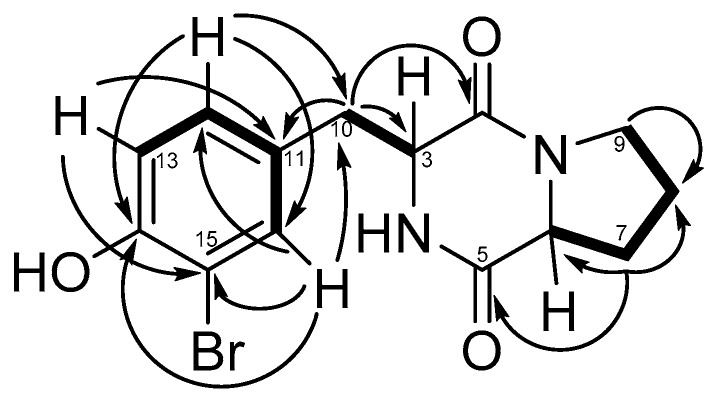
HMBC and COSY correlations on compound **7**.

The chemical shift of C-15 on ^13^C-NMR spectra and the characteristic isotopic pattern in the MS spectrum confirmed the presence of a bromide atom in the structure. First, ^1^H-NMR signals and chemical shift of H-3 and H-6 and the presence of two carbonyl functions (C-2 δ = 166.7, C-5 δ = 170.7) highlighted a diketopiperazine structure. Then, COSY correlations between the H-6, H-7, H-8, and H-9 protons established the presence of an alkyl chain. This chain was connected to a carbonyl function with an HMBC correlation between H-7 and C-5 corresponding to the proline group. Aromatic signals in ^1^H-NMR data (H-12, H-13, and H-16) were characteristic of a *meta*- *para*-disubstituted phenyl group, and chemical shifts of C-15 (δ: 154.5) and C-14 (δ: 110.6) showed the presence of a hydroxyl group and a bromide atom, respectively. COSY correlations between H-10 and H-3 and HMBC correlations between H-10 and C-3 and C-2 highlighted the linkage between the aromatic ring and the diketopiperazine moiety. Finally, the structure of compound **7** was established as shown in Figure 3.

Compound **6** was also obtained as a white amorphous powder, and its molecular formula was similar to that of compound **7**. Its ^1^H-NMR spectrum displayed similar patterns with those reported for compound **7**, but with variations in the chemical shifts—especially for H-3, H-6, H-7, and H-10 (Table 1). These data indicated that these compounds are two different diastereoisomers. To describe the stereochemistry of compounds **6** and **7**, their syntheses were realized.

#### 2.2.2. Synthesis

**Scheme 1:**
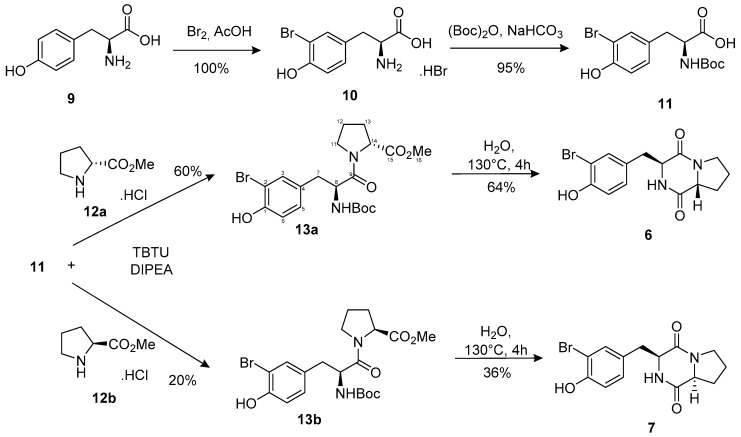
Synthesis of cyclo (d-Pro-l-Br-Tyr) (**6**) and cyclo (l-Pro-l-Br-Tyr) (**7**). TBTU: (*O*-(1*H*-Benzotriazol-1-yl)-*N*,*N*,*N*′,*N*′-tetramethyluronium tetrafluoroborate), DIPEA: (*N*,*N*-Diisopropyléthylamine).

The synthesis of the cyclo (l-Pro-l-Br-Tyr) **7** and one of its diastereoisomer, cyclo (d-Pro-l-Br-Tyr) **6** started with the bromination of l-tyrosine with Br_2_ in acidic condition. Next, the amine function was protected as its *N*-Boc derivative in the presence of sodium hydrogen carbonate to furnish intermediate **11** in an excellent yield over two steps. The *N*-Boc-protected l-Br-Tyrosine (**11**) was then condensed either with the l-Proline or the d-Proline in the presence of TBTU (*O*-(1*H*-Benzotriazol-1-yl)-*N*,*N*,*N*',*N*'-tetramethyluronium tetrafluoroborate) as the coupling reagent and the Hünig’s base DIPEA (*N*,*N*-Diisopropylethylamine) to yield **13a** (60%) and **13b** (20%), respectively. Finally, ring-closure was carried out in hot water at 130 °C during 4 h, affording compounds **6** and **7** in moderate 64% and 36% yields, respectively. The synthesis of **6** and **7** is outlined in Scheme 1.

^1^H-NMR data of the cyclo (d-Pro-l-Br-Tyr) and the cyclo (l-Pro-l-Br-Tyr) obtained in this way were similar to those from isolated compounds **6** and **7**, confirming the stereochemistry of these products. The presence of an amide bond was highlighted by the appearance of an N–H signal at 9.54 ppm for compound **6** in C_5_D_5_N and 8.72 ppm for compound **7** in (CD_3_)_2_CO.

### 2.3. Cytotoxic Activity

The cytotoxic activity of the two new synthesized diketopiperazines was evaluated against two different cell lines: HaCaT (human keratinocyte) and B16 (murine melanoma). For both compounds, the IC_50_ was >200 µg/mL.

Because the intermediates of synthesis **13a** and **13b** are newly described in this paper, their cytotoxic activity were also determined. Only one of them, **13b**, showed an interesting activity against the two cell lines HaCaT (IC_50_ 7 ± 2.5 µg/mL) and B16 (IC_50_ 18 ± 5 µg/mL).

The cytotoxicity of the six known compounds was been evaluated on both cell lines. Diketopiperazines (**1**–**5**) showed an IC_50_ > 200 µg/mL, as already described in a previous study for a mixture of diketopiperazines [16]. Indole-carboxaldehyde (**8**) had a weak cytotoxic activity on HaCaT (IC_50_ 79 ± 6 µg/mL) and B16 (IC_50_ 72 ± 6 µg/mL). All data are shown in Table 2.

## 3. Materials and Methods

### 3.1. General Experimental Procedures

All commercial reagents were purchased from Carlo Erba Reactifs and/or from Sigma Aldrich (Val de Reuil, France and St. Quentin Fallavier, France). An EasyPure (Barnstead™, ThermoFisher Waltham, MA, USA) water purification system was used to obtain HPLC and LC/MS grade water for chromatographic analysis. Deuterated solvents were purchased from Euriso-top (Gif-sur-Yvette, France). All spectra were recorded on a Bruker DMX 300 spectrometer (300 MHz (^1^H) and 75 MHz (^13^C)) and Bruker 500 cryo-spectrometer (500 MHz (^1^H) and 125 MHz (^13^C), Bruker, Billerica, MA, USA) using adequate deuterium solvents. Chemical shift values were referenced to residual solvent signals for CDCl_3_ (δ_H_/δ_C_, 7.26/77.16) and CD_3_OD (δ_H_/δ_C_, 3.31/49.00). HSQC, HMBC, COSY, or TOCSY data were recorded using a Bruker DMX 500 cryo-spectrometer instrument. NMR data were processed using the MestReNova version 9.1 software (Mestrelab Research, Santiago de Compostela, Coruña, Spain). The systems used for exact mass determination was an HPLC U3000 dual gradient RSLC (Rapid Separation Liquid Chromatography) coupled with a Q-Exactive Focus (LC-FT-MS/MS with a HESI probe), at Platform Bio2mar, Banyuls-sur-mer, France and an Agilent 6510 Q-TOF at CRMPO (Centre Regional de Mesures Physiques de l’Ouest), Rennes, France.

### 3.2. Microorganism

*Nocardia ignorata,* DP94 was isolated from the lichen *Collema auriforme* collected in Kesselfallklamm in Austria (47°12’21.26” N, 15°23’57.27” E) in November 2012. The strain was identified by sequencing its 16S rRNA gene using Sanger sequencing [2]. These data were compared with sequences in the Eztaxon server type strain database [17], and showed that the close phylogenetic neighbor of the strain was *Nocardia ignorata* DQ659907 at 98.59% sequence identity. The bacteria were stored after growth in ISP2 medium (International Streptomyces Project 2 medium) (4 g yeast extract (Sigma-Aldrich, St. Louis, MO, USA), 10 g malt extract (Sigma-Aldrich, St. Louis, MO, USA), and 4 g Dextrose (Sigma-Aldrich, St. Louis, Missouri) for 1 L) with 50% *v*/*v* glycerol or 5% *v*/*v* DMSO at −80 °C and referenced as DP94 (PNSCM collection).

### 3.3. Fermentation of *Nocardia ignorata* DP94

DP94 was cultivated in 50 mL test tubes containing 30 mL of TY medium (10 g yeast extract, Sigma-Aldrich, St. Louis, MO, USA), 16 g tryptone (Sigma-Aldrich, St. Louis, MO, USA), and 5 g NaCl (Sigma-Aldrich) for 1 L. The test tube was shaken on an orbital shaker New Brunswick Innova 42^®^ (110 rpm) at 25 °C for 72 h. Five liters of liquid TY medium were then inoculated with 60 mL of the test tubes culture in bioreactor Bioflo 115 (7.5 L, New Brunswick, Edison, NJ, USA). Growth parameters were fixed at 25 °C, aeration 0.4 vvm, dissolved oxygen percentage (DO) at 40% and stirring in cascade mode with DO (limited between 200 and 400 rpm), and pH was not controlled. After 4 days of culture, the medium was centrifuged at 3500 rpm (Thermo scientific Sorvall ST40R, ThermoFisher scientific, Waltham, MA, USA) during 15 min at 4 °C. The bacterial residue was frozen at −18 °C before further use. Forty grams of sterile resin XAD-7-HP (Sigma-Aldrich) were added for 1 L of supernatant medium before being shaken during 4 h using the same conditions (25 °C, 110 rpm).

### 3.4. Mass Spectrometry Analysis

Mass spectrometry analysis were carried out on a HPLC system—Diode Array Detector (LC-DAD) (Shimadzu, Marne La Vallée, France) and a mass spectrometer with a single quadrupole analyzer (Advion expression CMS, Ithaca, NY, USA). Ionization was made by electrospray in negative or positive mode (ESI) for low resolution analysis. High resolution analysis were performed either on HPLC U3000 dual gradient RSLC (Rapid Separation Liquid Chromatography) coupled with a Q-Exactive Focus (LC-FT-MS/MS with a HESI probe) or on Agilent 6510 Q-TOF mass spectra as already described. A Prevail C_18_ column (5 µm, 250 × 4.6 mm, GRACE, Columbia, MD, USA) was used for HPLC, and a gradient system was applied: A (0.1% formic acid in water) and B (0.1% formic acid in acetonitrile). The following gradient was applied at a flow rate of 0.8 mL/min in the HPLC system: initial: 100% (A); from 0 to 5 min: 100% (A); from 5 to 35 min: 100% (A)/0% (B) to 0% (A)/100% (B); from 35 to 45 min: 100% B; from 45 to 50 min: 100% (A)/0% (B) to 0% (A)/100% (B); from 50 to 55 min: 100% (A). A split to 0.2 mL/min was applied before mass spectrometry system. Twenty microliters were injected.

The Xcalibur 1.0 software was used for data analyses.

### 3.5. Extraction and Isolation

After filtration of the medium, the resin was extracted three successive times with acetone/MeOH (50/50, *v*/*v*) and the medium filtrate was kept for further use. Acetone/MeOH extracts were dried in vacuo*,* redissolved, and three further successive extractions with EtOAc/H_2_O (1/1, *v*/*v*) were realized. The organic phase (EtOAc extract) was collected and dried on anhydrous sodium sulfate. The organic phase was evaporated under vacuum to yield 657.3 mg of resin extract (RE). The medium previously depleted with the resin was extracted two times with EtOAc (1/2, *v*/*v*) in order to collect compounds not adsorbed on resin. The organic layer was desiccated with anhydrous sodium sulfate and dried under vacuum giving 137.6 mg of this supernatant extract (SE). These two extracts (resin and supernatant) were fractionated using various methods (Flash liquid chromatography (Puriflash Interchim) and semi-preparative HPLC).

For Flash liquid chromatography of the supernatant extract, the stationary phase was a C_18_ reversed phase pre-packed column (Chromabond Flash 25, 26 g, Macherey-Nagel, Hoerdt, France), and the mobile phase was a gradient: H_2_O (A)/acetonitrile (B) (80:20 to 100:0 in 190 min). Fourteen fractions were collected, and the fifth fraction (16.5 mg) was purified using semi-preparative HPLC with a Prevail C_18_ column and a gradient of water and acetonitrile (70:30). Five fractions were obtained: compound **6** in the second fraction and compound **7** in the third fraction.

For the resin extract, the flash chromatography was realized with a silica column pre-packed normal phase (SiO_2_) as stationary phase, and the mobile phase was a gradient: cyclohexane (A)/CH_2_Cl_2_ (B) (100:0 to 0:100 in 60 min), CH_2_Cl_2_ (B)/EtOAc (C) fractions (100:0 to 0:100 in 315 min) and EtOAc (C)/MeOH (D) (100:0 to 80:20 in 50 min) with a 10-min run per step. Twenty-three fractions were collected.

The sixth fraction (12.1 mg) was purified by semi-preparative HPLC with a Prevail C_18_ column and a gradient of H_2_O/acetonitrile (5 min 30% of B, 5 to 25 min 30:70 to 0:100, 25 to 30 min 100% B), yielding compound **8**.

The eighth fraction (235.3 mg) was firstly fractionated by flash chromatography using a silica gel column pre-packed normal phase (SiO_2_) and a gradient of cyclohexane, CH_2_Cl_2_, EtOAc, and MeOH in 150 min with a 10 min run per step. Ten fractions were collected, and compound **5** was purified from the fourth fraction by semi-preparative HPLC on a Prevail C_18_ column with a gradient of H_2_O (A)/acetonitrile (B) (100:0 to 40:60 in 20 min and 40:60 to 0:100 in 2 min and 100% B in 10 min). Compound **4** was purified from the sixth fraction by semi-preparative HPLC on a Prevail™ C_18_ column with a gradient of H_2_O/acetonitrile 70:30.

The ninth fraction (143.1 mg) was also fractionated by flash chromatography using a silica gel column pre-packed normal phase (SiO_2_) and a gradient of cyclohexane, CH_2_Cl_2_, EtOAc, and MeOH in 150 min with a 10-min run per step. Eleven fractions were collected, and compound **3** was purified from the third fraction by semi-preparative HPLC with the Prevail C_18_ column with a gradient of H_2_O/acetonitrile 70:30. Compound **2** was purified from the seventh fraction with the same system (Prevail C_18_ column) and a gradient of H_2_O/acetonitrile 75:25.

The 21st fraction (6.9 mg) afforded compound **1**.

### 3.6. Biological Assays: Cytotoxic Activities

Cytotoxic properties of pure compounds were determined with a standard tetrazolium assay [18]. B16 (6000 cells/well) and HaCaT (10,000 cells/well) cells were seeded in RPMI 1640 medium with 5% fetal calf serum (FCS) and antibiotic at day 0 in well-plate. Incubation was performed at 37 °C in an atmosphere of 5% CO_2_. After 24 h of incubation, compounds were added at different concentrations (1, 10, 50, 100, and 200 µg/mL), and the plate was incubated 24 h more (37 °C, 5% CO_2_). Cell growth and viability were then measured at 540 nm, using a MTT (3-[4,5-dimethylthiazol-2-yl]-2,5-diphenyltetrazolium bromide) assay. Each experiment was repeated at least three times.

### 3.7. Preparation of l-Bromo-Tyrosine *(**10**)*

l-Tyrosine (5.0 g, 55.0 mmol) was dissolved into 15 mL of acetic acid. A solution of bromine (4.3 g, 55.0 mmol) solubilized in 3 mL of acetic acid was added dropwise and the reaction mixture was stirred at room temperature for 4 h. The precipitate was then filtrated and washed with acetic acid and diethyl ether, affording **10** as a white solid. This compound was used in the next step without further purification.

*3-Bromo-l-tyrosine* (**10**): white amorphous powder, ^1^H-NMR (CD_3_OD, 300 MHz), and ^13^C-NMR data (CD_3_OD, 75 MHz) as described in the literature [19].

### 3.8. Protection of the Amine Group *(**11**)*

The brominated amino acid (**10**) (1 eq.) (4.5 g, 13.2 mmol) was poured in 60 mL of a mixture of MeOH/acetone (1:1). A solution of sodium hydrogen carbonate (2.2 eq.) (2.4 g, 29.3 mmol) in 20 mL of water and 1.6 eq. of (BOC)_2_O (4.8 g, 22.0 mmol) were added successively to the reaction medium after stirring at room temperature for 4 h. The solvent was removed under vacuum, and the aqueous residue was washed with cyclohexane and acidified with citric acid until reaching pH 2. The product precipitated and was extracted from the aqueous layer with 3 × 30 mL of ethyl acetate. The organic layer was then dried with Na_2_SO_4_, and the solvent was removed in vacuo leading to 4.5 g (95%) of the protected product (**11**).

*N-Boc-3-Bromo-l-tyrosine* (**11**): white amorphous powder, ^1^H-NMR and ^13^C-NMR data (MeOD, 75 MHz) as described in the literature [19].

### 3.9. Condensation of the Amino Acids

General procedure: To a solution of intermediate **11** in (Dichloromethane/acetonitrile) (9/1) was added successively 1.0 eq. of (d or l) proline methyl ester hydrochloride (**12a** or **12b**), 2.2 eq. of DIPEA and 1.2 eq. of TBTU. After stirring at room temperature for 2 h, the reaction was quenched by adding NH_4_Cl and then washed with a solution of saturated NaHCO_3_ and brine. The organic residue was then dried with Na_2_SO_4_ and concentrated under vacuum. The crude product was purified on silica gel using CH_2_Cl_2_/EtOAc (60:40) as eluent to afford the final products **13a** (l-Tyr, d-Pro) (60%) and **13b** (l-Tyr, l-Pro) (20%).

*Methyl ((S)-3-(3-bromo-4-hydroxyphenyl)-2-((tert-butoxycarbonyl)amino)propanoyl)-d-prolinate* (**13a**): prepared from **11** (0.76 g, 2.1 mmol) and **12a** (0.35 g, 2.1 mmol) in 20 mL of solvent (DIPEA 0.60 g, 4.6 mmol; TBTU 0.81 g, 2.5 mmol); white oil; ${\left[\alpha \right]}_{D}^{20}$ −0.11 (*c* 0.77, CHCl_3_), ^1^H-NMR (300 MHz, CDCl_3_) δ 7.28 (d, *J* = 2.1 Hz, 1H, H-3), 7.04 (dd, *J* = 8.3, 2.1 Hz, 1H, H-5), 6.86 (d, *J* = 8.3 Hz, 1H, H-6), 5.42 (d, *J* = 8.5 Hz, 1H, NH-Boc), 4.56 (dd, *J* = 7.3, 6.3 Hz, 1H, H-8), 4.33 (dd, *J* = 8.0, 3.8 Hz, 1H, H-14), 3.71 (s, 3H, O-CH_3_), 3.55–3.65 (m, 1H, H-11a), 3.03–2.71 (m, 3H, H-11b, H-7a, H-7b), 2.01–1.79 (m, 3H, H12a, H-12b, H-13a), 1.63 (m, 1H, H-13b), 1.41 (s, 9H, Boc C-(CH_3_)_3_), ^13^C-NMR (75 MHz, CDCl_3_) δ 172.2 (C-15), 170.1 (C-9), 155.0 (Boc C = O), 151.3 (C-1), 132.8 (C-3), 130.4 (C-4), 130.2 (C-5), 115.9 (C-6), 109.8 (C-2), 79.9 (Boc C-(CH_3_)_3_), 58.8 (C-14), 53.5 (C-8), 52.3 (C-16), 46.9 (C-11), 38.9 (C-7), 29.0 (C-12), 28.3 (Boc C-(CH_3_)_3_), 24.5 (C-13), HRESIMS *m*/*z* 493.0945 [M + Na]^+^ (calcd. for C_20_H_26_BrN_2_O_6_Na, 493.0945).

*Methyl ((S)-3-(3-bromo-4-hydroxyphenyl)-2-((tert-butoxycarbonyl)amino)propanoyl)-l-prolinate* (**13b**): prepared from **11** (1.8 g, 5.0 mmol) and **12b** (0.83 g, 5.0 mmol) in 50 mL of solvent (DIPEA 1.42 g, 11.0 mmol; TBTU 1.90 g, 5.9 mmol); white oil; ${\left[\alpha \right]}_{D}^{20}$ +0.39 (*c* 0.77, CHCl_3_), ^1^H-NMR (500 MHz, CDCl_3_) mixture of rotamers δ 7.55 and 7.37 (d, *J* = 2.1 Hz, 1H, H-3), 7.25 and 7.10 (dd, *J* = 8.3, 2.2 Hz, 1H, H-5), 7.04 and 6.89 (d, *J* = 8.3 Hz, 1H, H-6), 4.82 and 4.60 (q, *J* = 6.6 Hz, 1H, H-8), 4.40 and 4.50 (dd, *J* = 8.4, 4.1 Hz, 1H, H-14), 3.62 and 3.75 (s, 3H, O-CH_3_), 3.40, 3.21 and 3.67–3.55 (m, 2H, H-11a and H-11b), 3.35 and 3.02 (dd, *J* = 13.9, 6.7 Hz, 1H, H-7a), 3.17 and 2.82 (dd, *J* = 13.9, 5.9 Hz, 1H, H-7b), 2.18 and 2.05 (m, 1H, H-12a), 1.96 and 1.81 (m, 1H, H-12b), 2.02–1.88 and 1.74 (m, 2H, H-13a and H-13b), 1.39 (s, 9H, Boc C-(CH_3_)_3_), ^13^C-NMR (125 MHz, CDCl_3_) δ 172.5 and 172.2 (C-15), 170.2 and 170.1 (C-9), 155.2 (Boc C = O), 148.5 and 148.2 (C-1), 134.7 and 133.1 (C-3), 129.7 (C-4), 130.3 and 130.1 (C-5), 123.3 and 116.4 (C-6), 109.8 and 109.7 (C-2), 80.1 (Boc C-(CH_3_)_3_), 59.2 and 53.1 (C-14), 54.6 and 52.8 (C-8), 52.5 and 52.4 (C-16), 47.2 (C-11), 38.3 and 37.3 (C-7), 30.7 and 29.2 (C-12), 28.4 (Boc C-(CH_3_)_3_), 25.0 and 22.4 (C-13), HRESIMS *m*/*z* 493.0941 [M + Na]^+^ (calcd. for C_20_H_26_BrN_2_O_6_Na, 493.0945).

### 3.10. Deprotection and Cyclization

Compounds **13a** (0.44 g, 0.9 mmol) and **13b** (0.23 g, 0.5 mmol)) were poured into a reactor with respectively 15 mL and 3 mL of water and heated to 130 °C during 4 h as described in literature for a one-pot deprotection and cyclization [20]. After cooling, the reaction mixture was extracted three times with 15 mL of ethyl acetate. The organic layer was desiccated with Na_2_SO_4_ and dried under vacuum. The residues were purified on silica gel using different eluent systems: 100% ethyl acetate for compound **13a** and dichloromethane/ethyl acetate (60:40) for compound **13b**. These purifications yielded to the final products **6** (64%) and **7** (36%).

### 3.11. Analytical Data

All fractions were analyzed with HPLC-UV using a Prevail C_18_ column with a gradient of H_2_O (A)/acetonitrile (B): 5 min 100:0, 30 min from 0% of B to 100% of B, 10 min 100% B followed by equilibration of the column.

*Cyclo (l-Pro-l-O-Met)* (**1**): 6.9 mg, white amorphous powder; rt: 18.8 min, ${\left[\alpha \right]}_{D}^{20}$ −4.0 (*c* 1, MeOH), ^1^H-NMR (CDCl_3_, 500 MHz) and ^13^C-NMR data (CDCl_3_, 125 MHz) as described in the literature [12], HRESIMS *m*/*z* 245.1277 [M + H]^+^ (calcd. for C_10_H_17_N_2_O_3_S, 245.0955).

*Cyclo (l-Pro-l-Tyr)* (**2**): 5.7 mg, white amorphous powder; r.t.: 20.7 min, ${\left[\alpha \right]}_{D}^{20}$ +15.7 (*c* 1, MeOH), ^1^H-NMR (CD_3_OD, 500 MHz) and ^13^C-NMR data (CD_3_OD, 125 MHz) as described in the literature [13,14], HRESIMS *m/z* 261.1225 [M + H]^+^ (calcd. for C_14_H_17_N_2_O_3_, 261.1234).

*Cyclo (d-Pro-l-Tyr)* (**3**): 8.5 mg, white amorphous powder; r.t.: 21.1 min, ${\left[\alpha \right]}_{D}^{20}$ −38.2 (*c* 1, MeOH), ^1^H-NMR (CD_3_OD, 500 MHz) and ^13^C-NMR data (CD_3_OD, 125 MHz) as described in the literature [13], HRESIMS *m*/*z* 261.1225 [M + H]^+^ (calcd. for C_14_H_17_N_2_O_3_, 261.1234).

*Cyclo (l-Pro-l-Val)* (**4**): 1.1 mg, white amorphous powder; r.t.: 21.9 min, ${\left[\alpha \right]}_{D}^{20}$ −23.0 (*c* 1, CHCl_3_), ^1^H-NMR (CD_3_OD, 500 MHz) and ^13^C-NMR data (CD_3_OD, 125 MHz) as described in the literature [14], HRESIMS *m*/*z* 197.1282 [M + H]^+^ (calcd. for C_10_H_17_N_2_O_2_, 197.1285).

*Cyclo (l-Pro-l-Leu)* (**5**): 4.3 mg, white amorphous powder; r.t.: 24.1 min, ${\left[\alpha \right]}_{D}^{20}$ −27.4 (c 1, MeOH), ^1^H-NMR (CD_3_OD, 500 MHz) and ^13^C-NMR data (CD_3_OD, 125 MHz) as described in the literature [14,15], HRESIMS *m*/*z* 211.1438 [M + H]^+^ (calcd. for C_11_H_19_N_2_O_2_, 211.1441).

*Cyclo (d-Pro-l-Br-Tyr)* (**6**): 1.4 mg, white amorphous powder; r.t.: 23.4 min, ${\left[\alpha \right]}_{D}^{20}$ +38.4 (*c* 0.77, MeOH), ^1^H-NMR (CD_3_OD, 500 MHz) and ^13^C-NMR data (CD_3_OD, 125 MHz) as described in Table 1, HRESIMS *m*/*z* 339.0329 [M + H]^+^ (calcd. for C_14_H_16_BrN_2_O_3_, 339.0339).

*Cyclo (l-Pro-l-Br-Tyr)* (**7**): 2.1 mg, white amorphous powder; r.t.: 23.9 min, ${\left[\alpha \right]}_{D}^{20}$ −29.4 (*c* 0.77, MeOH), ^1^H-NMR (CD_3_OD, 500 MHz) and ^13^C-NMR data (CD_3_OD, 125 MHz) as described in Table 1, HRESIMS *m*/*z* 339.0329 [M + H]^+^ (calcd. for C_14_H_16_BrN_2_O_3_, 339.0339).

*Indole-Carboxaldehyde* (**8**): 5.0 mg, white amorphous powder; r.t.: 26.2 min, ^1^H-NMR (CD_3_OD, 500 MHz) and ^13^C-NMR data (CD_3_OD, 125 MHz) as described in the literature [15], HRESIMS *m*/*z* 146.0598 [M + H]^+^ (calcd. for C_9_H_8_NO, 146.0600).

## 4. Conclusions

In this study two new diketopiperazines as well as five known diketopiperazines and one known auxin derivative have been isolated from the culture medium of our strain *Nocardia ignorata*. The synthesis of the new compounds was realized, and confirmed the structure elucidated by 2D NMR data and HRMS. None of the compounds isolated—except the auxin derivative—presented cytotoxic activity, but these compounds are well known for playing a role in bacterial communication. The role of diketopiperazines in quorum sensing is currently described. These compounds could thus interact with *N*-acylhomoserine lactone (AHL) biosensor and in this way they can modulate bacterial characters such as bioluminescence (via LuxR from *Vibrio fischeri*) [21], swarming in *Serratia liquefaciens* [22], pathogenicity of diverse bacteria [23,24], and could inhibit bacterial biofilm [25]. These types of compounds involved in cell-to-cell communications activate specific bacterial behavior by regulating the gene expression in response to bacterial density [26]. The two new compounds—only produced during the death phase of the bacterial growth—probably have a role in decline of bacteria during this stage. Moreover, the production of brominated compounds from our bacterium highlights the presence of a halogenase in the enzymatic machinery of this strain.

## Figures and Tables

**Table 1:** ^1^H-NMR and ^13^C-NMR spectroscopic data of compounds **6** and **7**.

No.	6		7	
	δ_H_ (*J* in Hz)	δ_C_	δ_H_ (*J* in Hz)	δ_C_
**1**	-	-	-	-
**2**	-	167.3	-	166.7
**3**	4.14, t (4.7)	59.8	4.38, td (4.7, 2.0)	57.7
**4**	-	-	-	-
**5**	-	171.1	-	170.7
**6**	2.82, m	59.3	4.07, ddd (10.9, 6.3, 2.0)	60.0
**7**	1.70, m	29.9	1.25, m	29.5
	2.12, m		2.13, m	
**8**	1.95, m 1.70, m	22.5	1.84, m	22.6
**9**	3.36, m	46.2	3.38, m	45.9
	3.56, m		3.57, m	
**10**	2.88, dd (13.9, 4.9)	39.7	2.99, dd (14.2, 4.7)	37.1
	3.09, dd (13.9, 4.9)		3.13, dd (14.2, 4.7)	
**11**	-	128.9	-	129.6
**12**	6.98, dd (8.2, 2.1)	131.3	7.03, dd (8.3, 2.2)	131.4
**13**	6.83, d (8.2)	117.2	6.81, d (8.3)	116.9
**14**	-	110.8	-	110.6
**15**	-	155.0	-	154.5
**16**	7.29, d (2.1)	135.5	7.35, d (2.2)	135.4

**Table 2:** Cytotoxic activity of compounds **1**–**8** and **13a**–**b**.

Compounds		1	2	3	4	5	6	7	8	13a	13b
IC_50_ (µg/mL)	HaCaT	>200	>200	>200	>200	>200	>200	>200	79 ± 6	>200	7 ± 2.5
	B16	>200	>200	>200	>200	>200	>200	>200	72 ± 6	>200	18 ± 5

## Supplementary Materials

Supplementary materials are available online.
