# Supplementary material for: Isolation and Structure Identification of Novel Brominated Diketopiperazines from Nocardia ignorata—A Lichen-Associated Actinobacterium
Source: Molecules. 2017 Feb 28;22(3):371. doi: 10.3390/molecules22030371 (PMC6155340; doi:10.3390/molecules22030371)
Supplement: Supplementary file 1 [file molecules-22-00371-s001.pdf]

Isolation and Structure Identification of Novel Brominated Diketopiperazines from *Nocardia ignorata*—A Lichen-Associated Actinobacterium

Alba Noël [1], Solenn Ferron [1], Isabelle Rouaud [1], Nicolas Gouault [1], Jean-Pierre Hurvois [1] and Sophie Tomasi [1,*]

[1] CORINT, UMR CNRS ISCR 6226, UFR Sciences Pharmaceutiques et Biologiques, Université Bretagne Loire, 2 Av. du Professeur Léon Bernard, 35043 Rennes, France;

* Correspondence: sophie.tomasi@univ-rennes1.fr; Tel.: +33-223-234-817

Supplementary material

Figure S1: ^{1}H-NMR spectrum of compound **1** in CD_3OD 2
Figure S2: ^{1}H-NMR spectrum of compound **2** in CD_3OD 3
Figure S3 : ^{1}H-NMR spectrum of compound **3** in CD_3OD 4
Figure S4: ^{1}H-NMR spectrum of compound **4** in $CDCl_3$ 5
Figure S5: ^{1}H-NMR spectrum of compound **5** in $CDCl_3$ 6
Figure S6: ^{1}H-NMR spectrum of compound **6** in CD_3OD 7
Figure S7: ^{13}C-NMR spectrum of compound **6** in CD_3OD 8
Figure S8 : HMBC spectrum of compound **6** in CD_3OD 9
Figure S9: ^{1}H-NMR spectrum of compound **6** in pyridine-d_5 10
Figure S10 : HMBC spectrum of compound **6** in pyridine-d_5 11
Figure 11 : NOESY spectrum of compound **6** in pyridine-d_5 12
Figure S12: ^{1}H-NMR spectrum of compound **7** in CD_3OD 13
Figure S13: ^{13}C-NMR spectrum of compound **7** in CD_3OD 14
Figure S14: HSQC spectrum of compound **7** in CD_3OD 15
Figure S15: COSY spectrum of compound **7** in CD_3OD 16
Figure S16: HMBC spectrum of compound **7** in CD_3OD 17
Figure S17: ^{1}H-NMR spectrum of compound **7** in acetone-d_6 18
Figure S18 : HMBC spectrum of compound **7** in acetone-d_6 19
Figure S19: NOESY spectrum of compound **7** in acetone-d_6 20
Figure S20: ^{1}H-NMR spectrum of compound **8** in CD_3OD 21
Figure S21: ^{1}H-NMR spectrum of compound **10** in DMSO-d_6 22
Figure S22: ^{1}H-NMR spectrum of compound **11** in $CDCl_3$ 23
Figure S23: ^{1}H-NMR spectrum of compound **13a** in $CDCl_3$ 24
Figure S24: ^{13}C-NMR spectrum of compound **13a** in $CDCl_3$ 25
Figure S25: ^{1}H-NMR spectrum of compound **13b** in $CDCl_3$ 26
Figure S26: ^{13}C-NMR spectrum of compound **13b** in $CDCl_3$ 27
Figure S27: HPLC chromatogram at 220 nm of isolated (blue) and synthetic (red) compounds **6** (a) and **7** (b) 28

S1

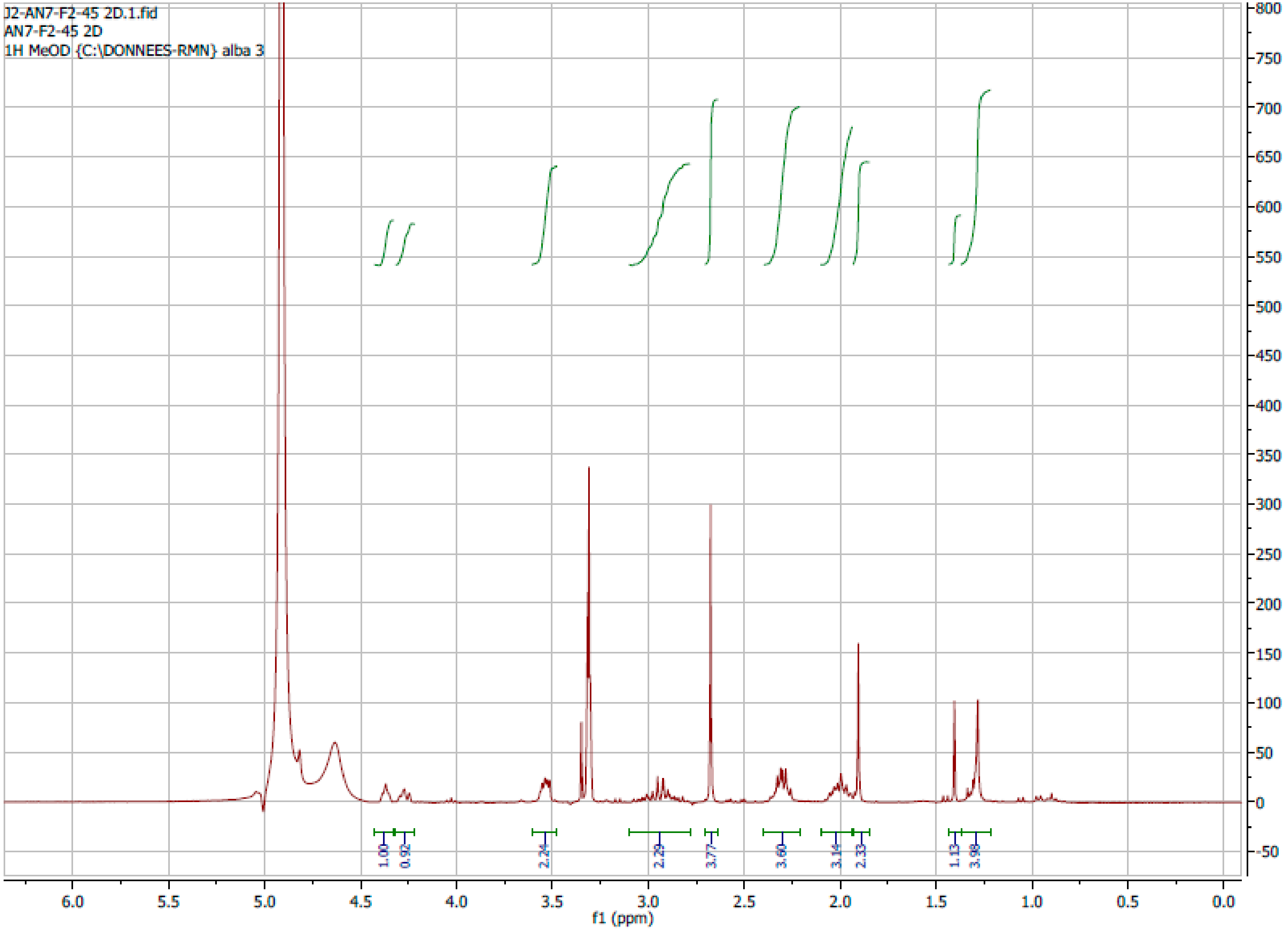

Figure S1: *^{1}H-NMR spectrum of compound* ***1*** *in* CD_3OD.

S2

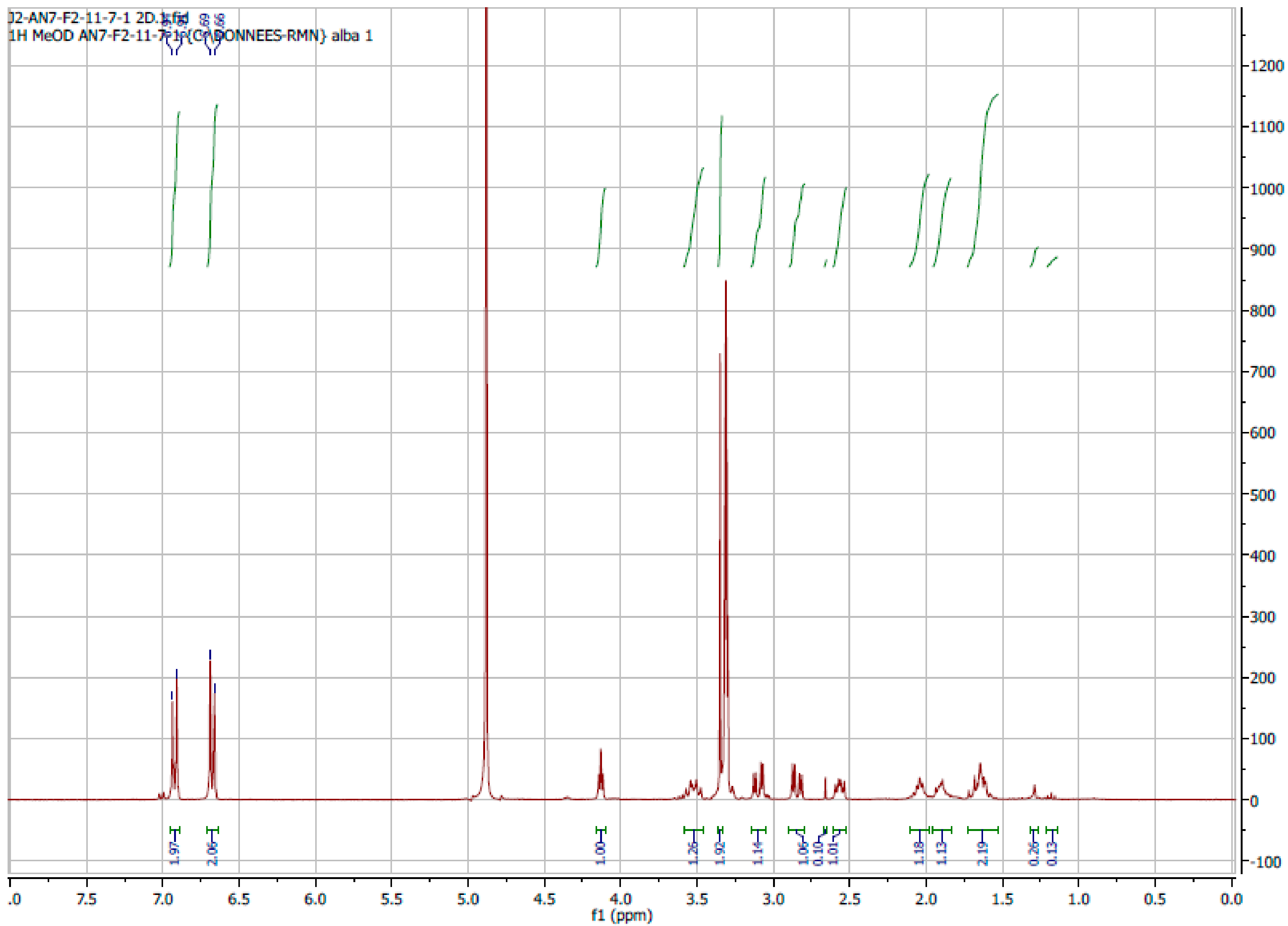

Figure S2: *^{1}H-NMR spectrum of compound* ***2*** *in CD₃OD.*

S3

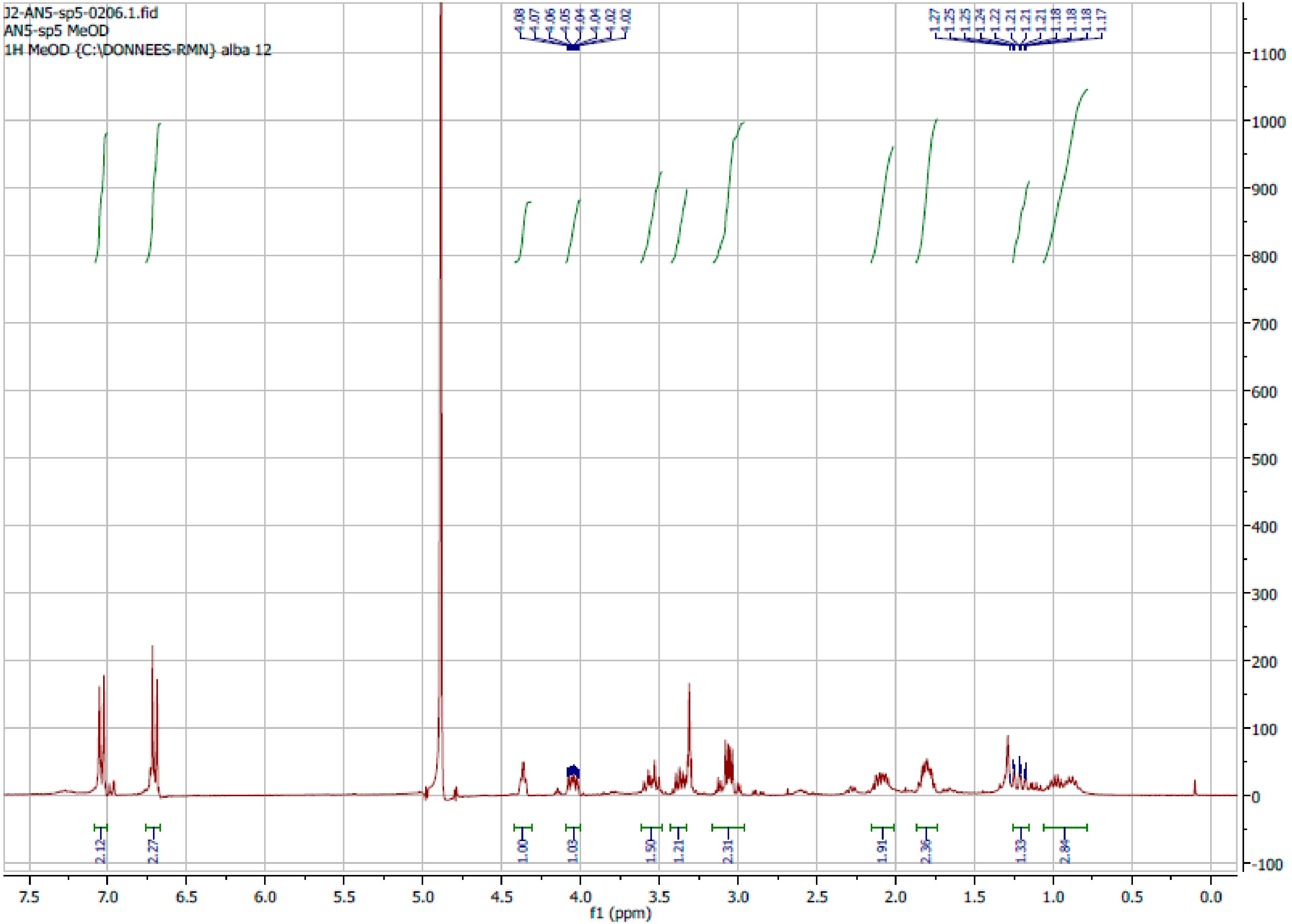

Figure S3 : *^{1}H-NMR spectrum of compound* ***3*** *in CD_3OD.*

S4

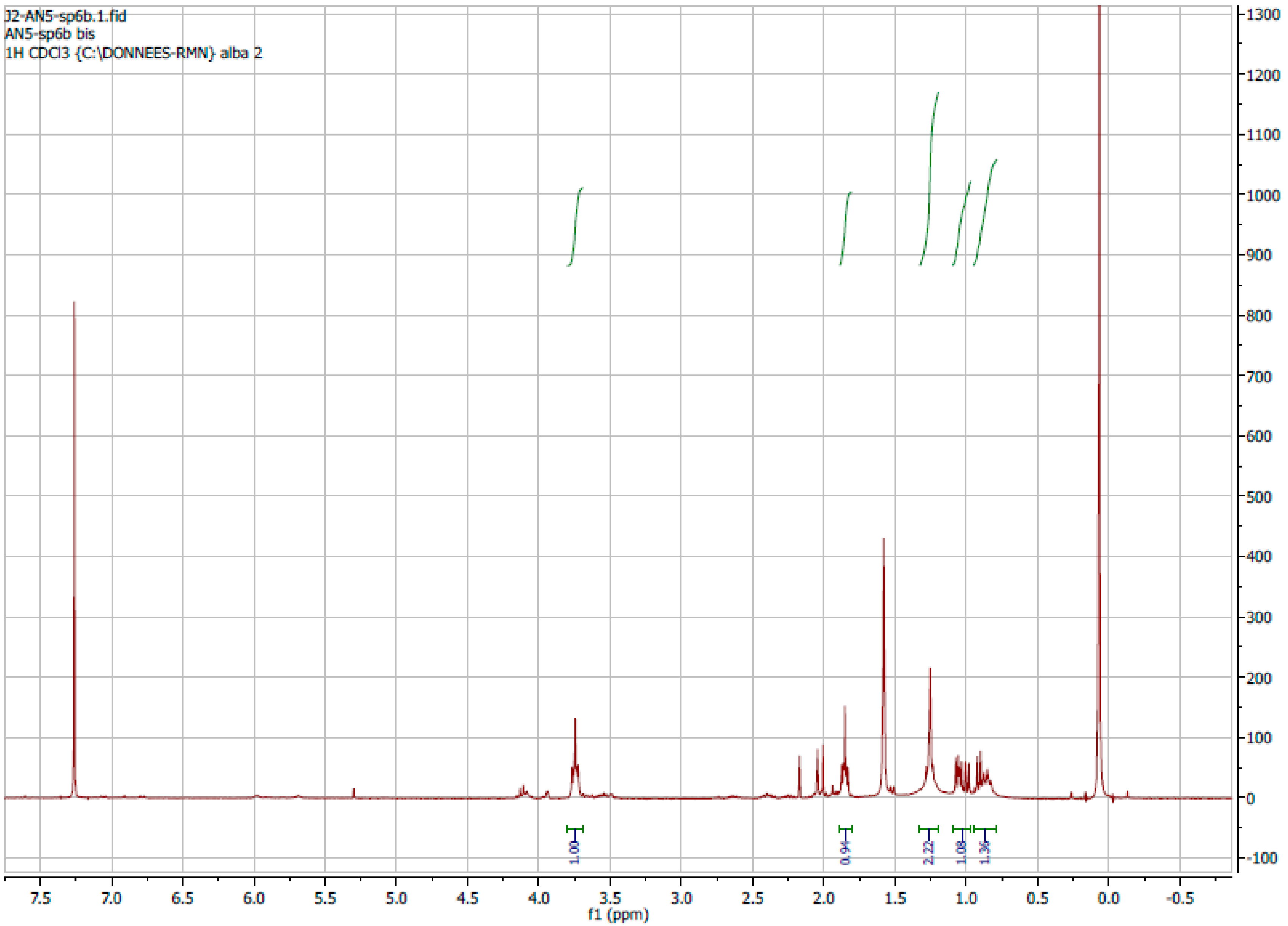

Figure S4: *^{1}H-NMR spectrum of compound* ***4*** *in CDCl₃.*

S5

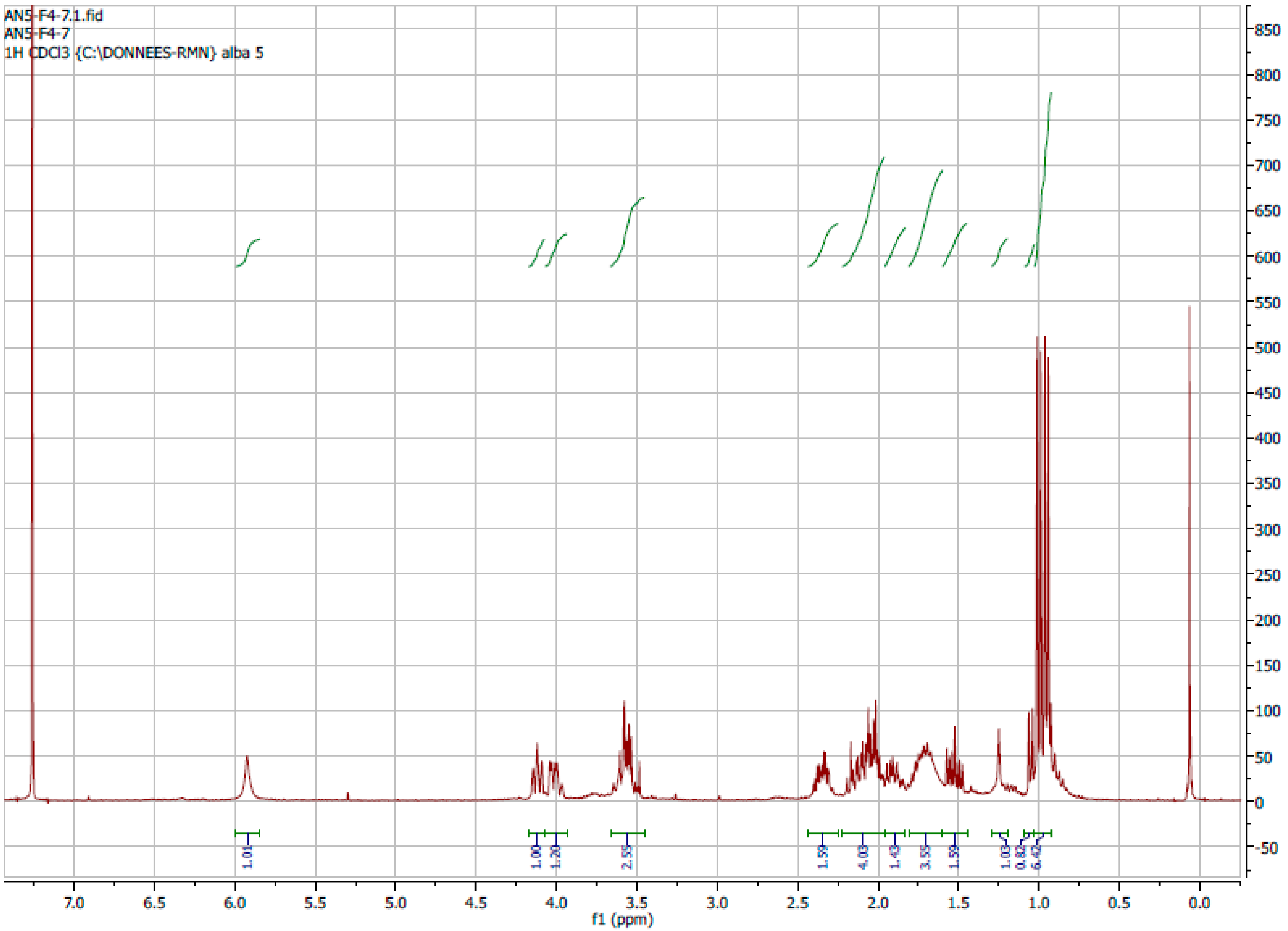

Figure S5: *^{1}H-NMR spectrum of compound **5** in $CDCl_3$.*

S6

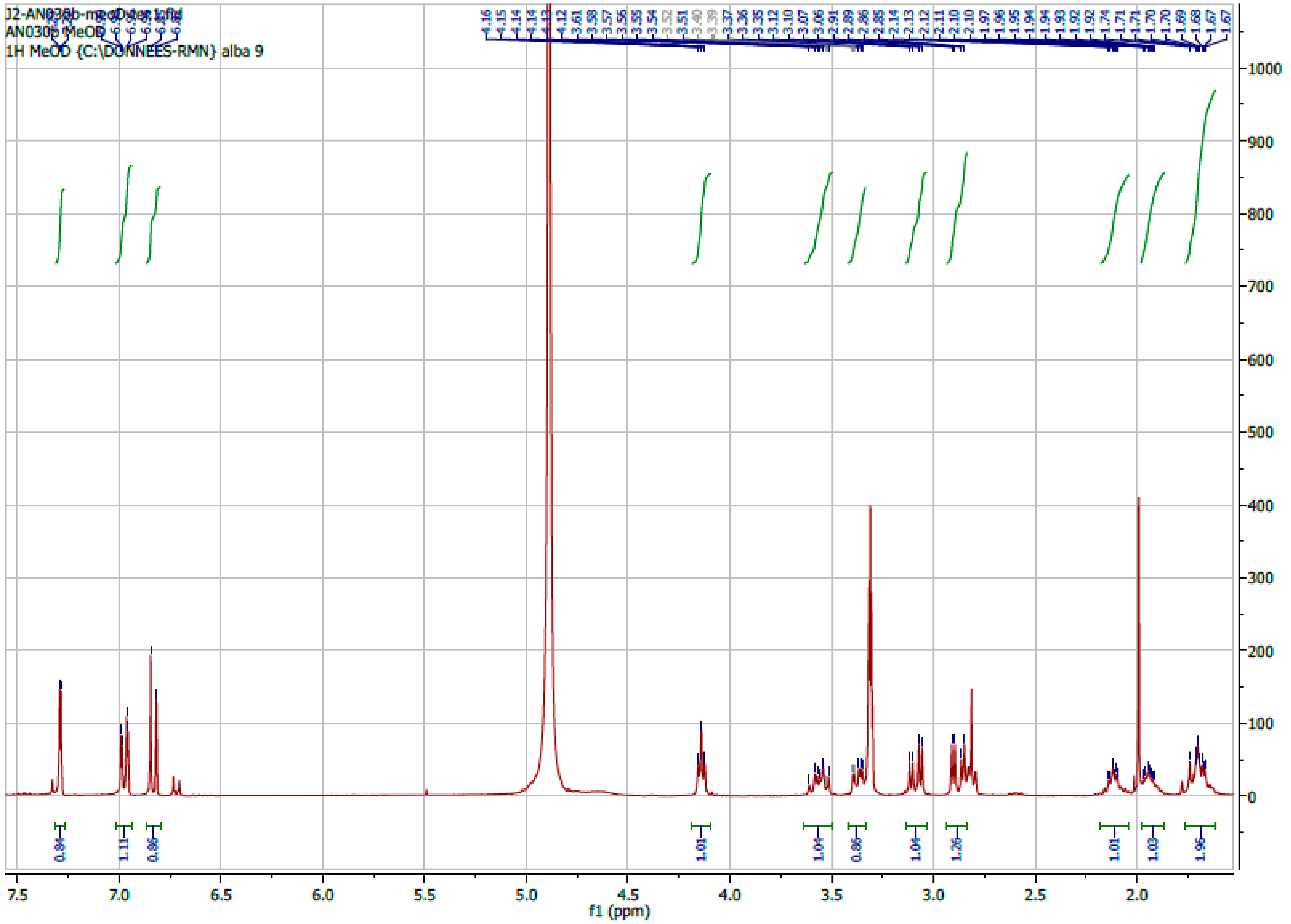

Figure S6: *¹H-NMR spectrum of compound* ***6*** *in CD₃OD.*

S7

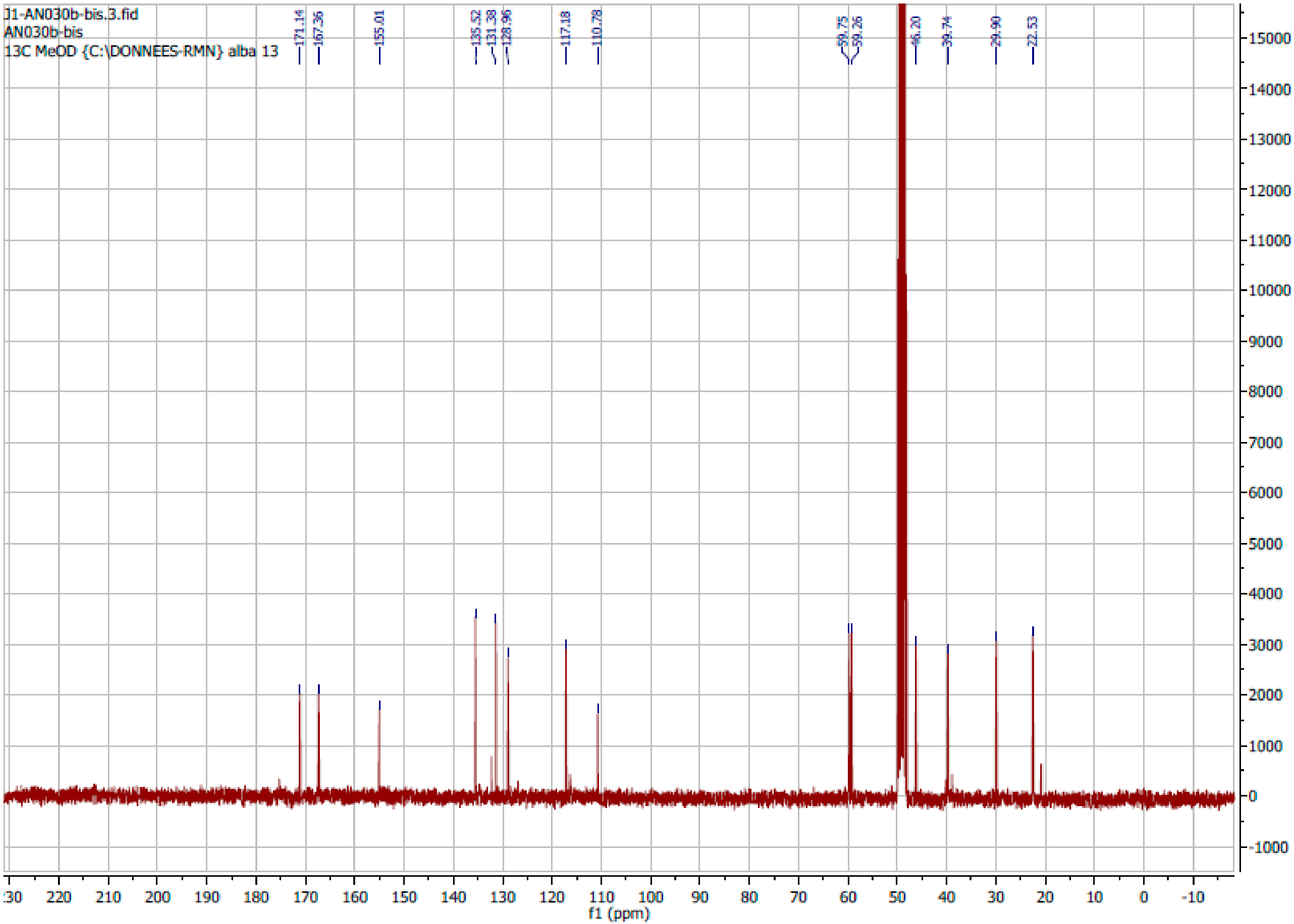

Figure S7: *^{13}C-NMR spectrum of compound* ***6*** *in CD_3OD.*

S8

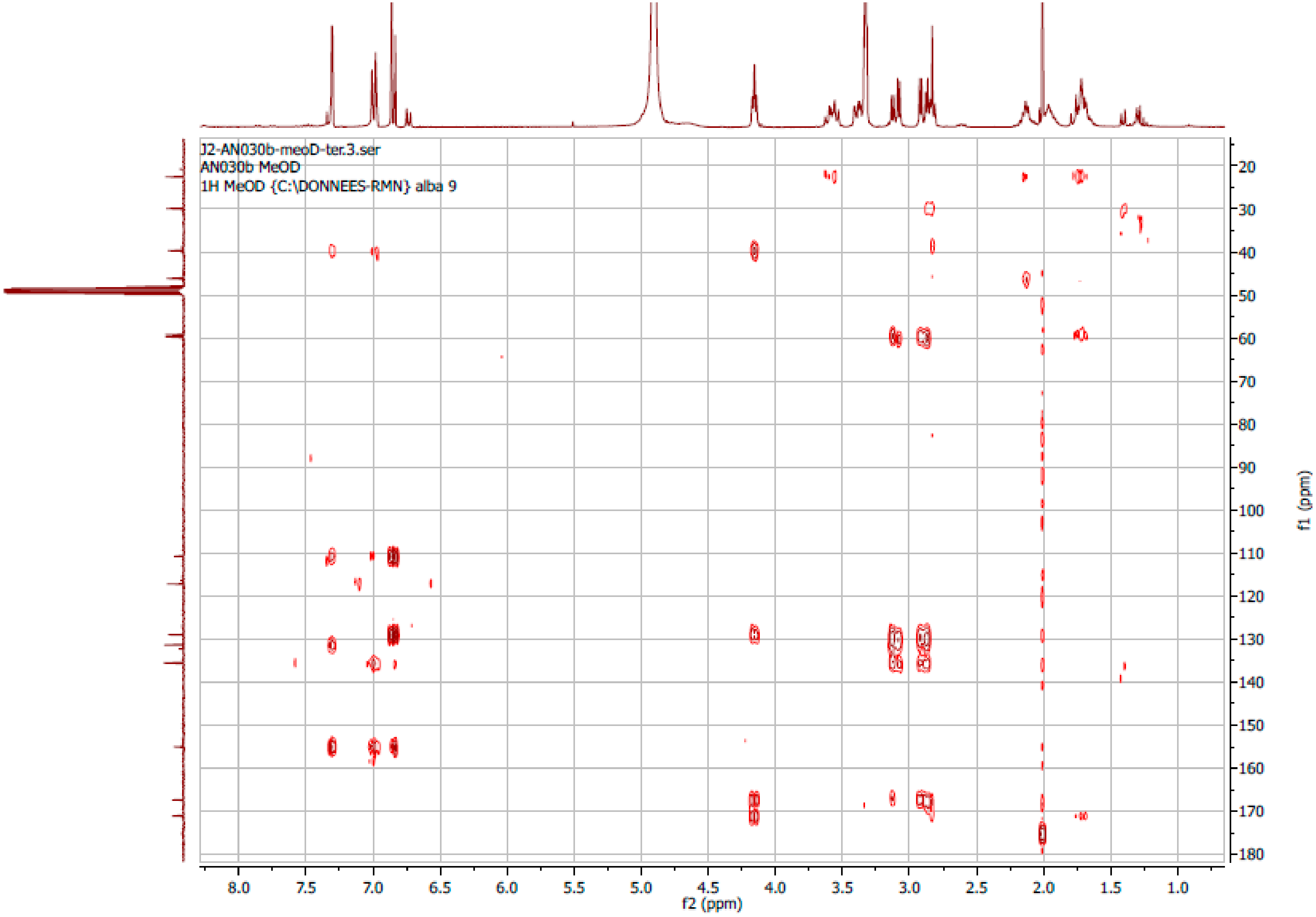

Figure S8 *: HMBC spectrum of compound **6** in CD_3OD.*

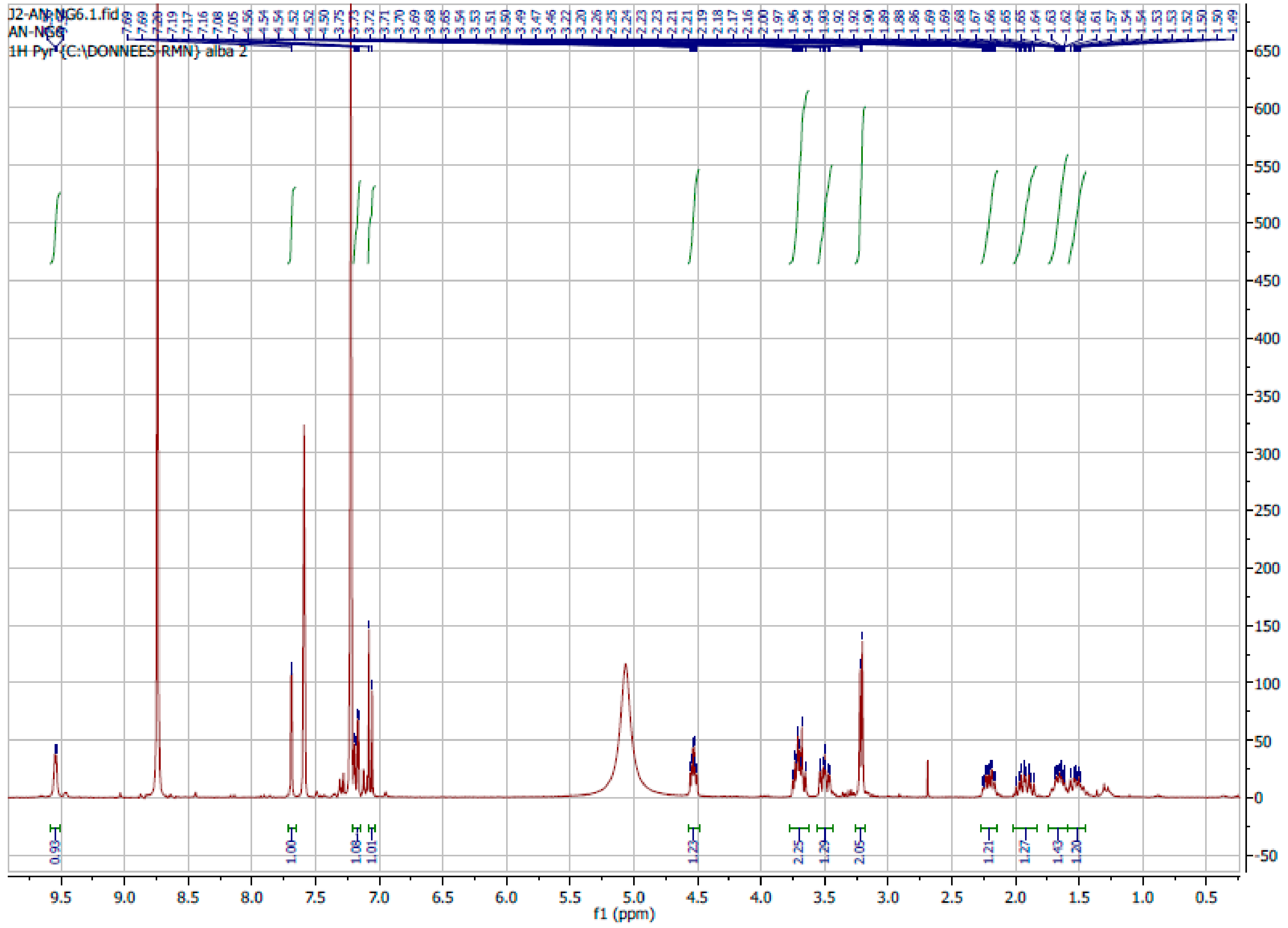

Figure S9: *^{1}H-NMR spectrum of compound* ***6*** *in pyridine-d5.*

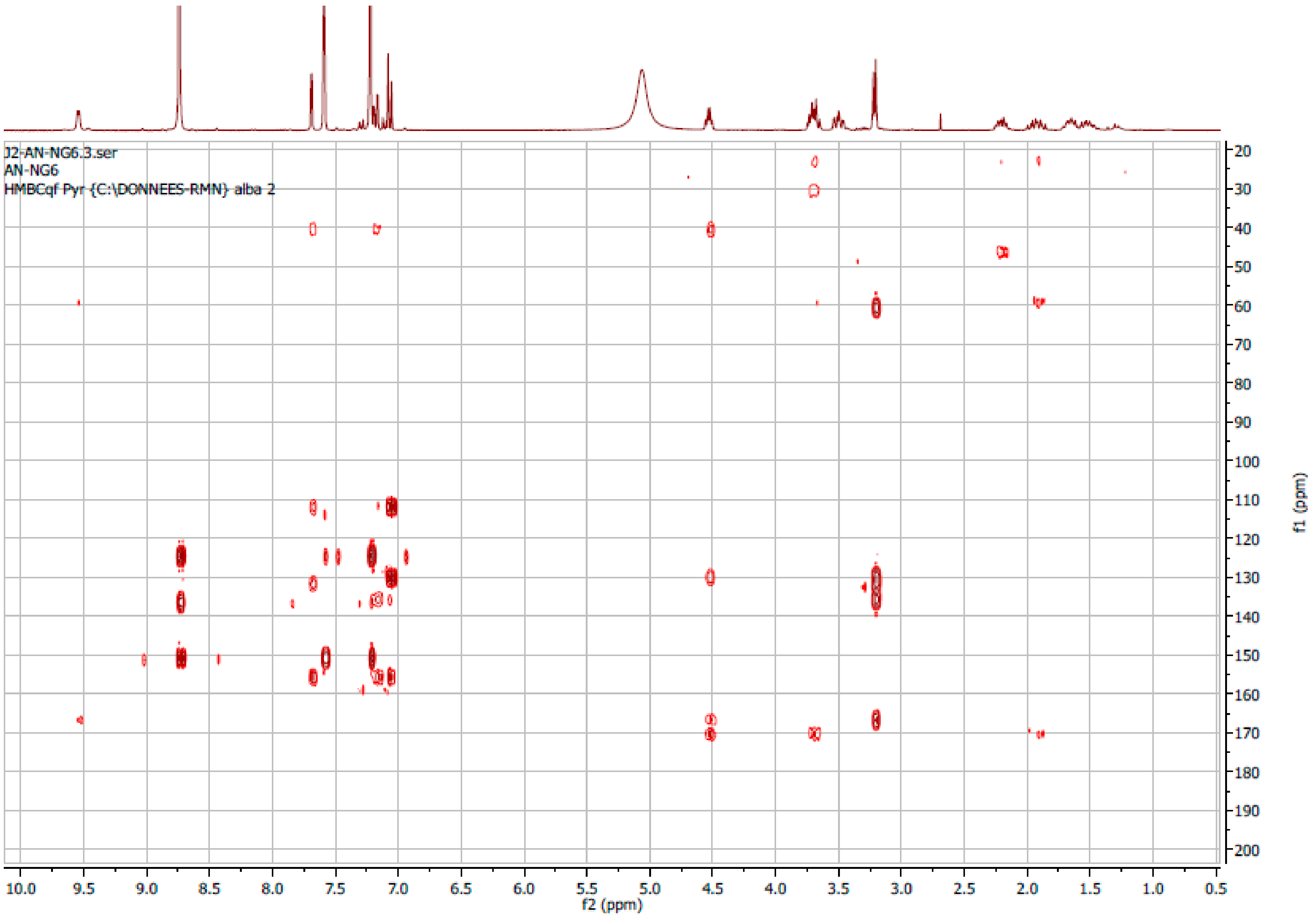

Figure S10 : *HMBC spectrum of compound **6** in pyridine-d5.*

S11

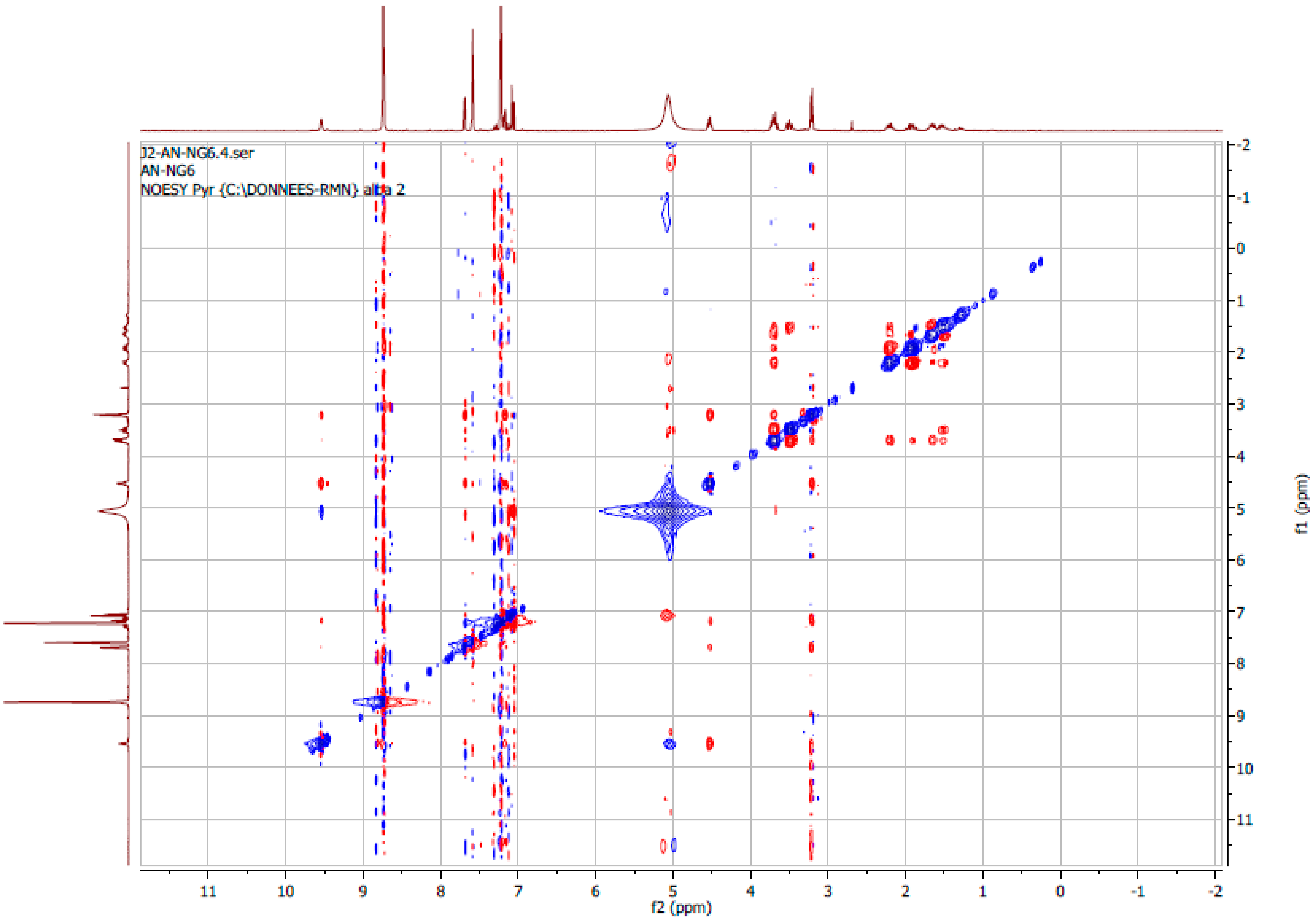

Figure S11 : *NOESY spectrum of compound* ***6*** *in pyridine-d5.*

S12

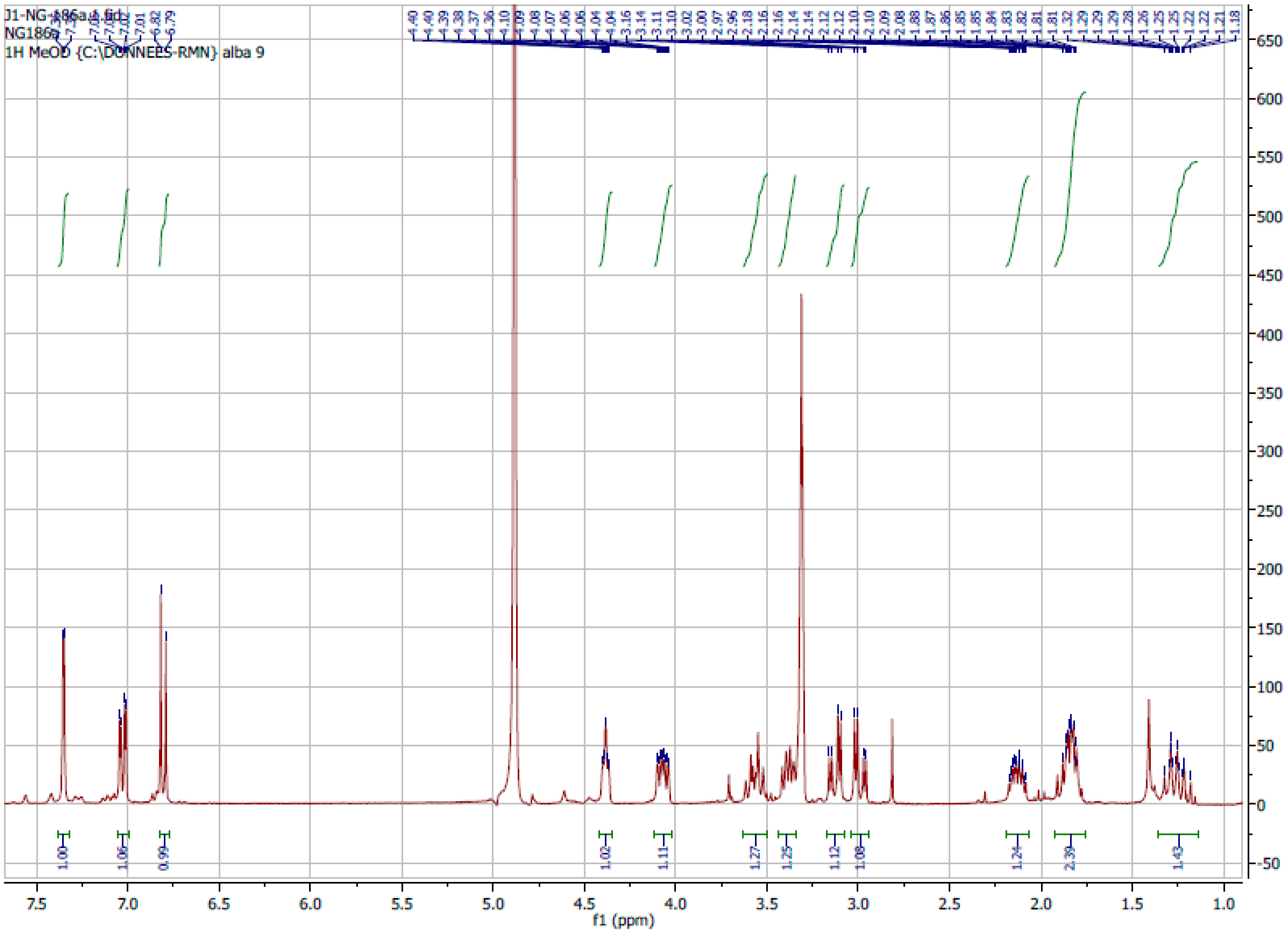

***Figure S12**: ^{1}H-NMR spectrum of compound **7** in CD_3OD.*

S13

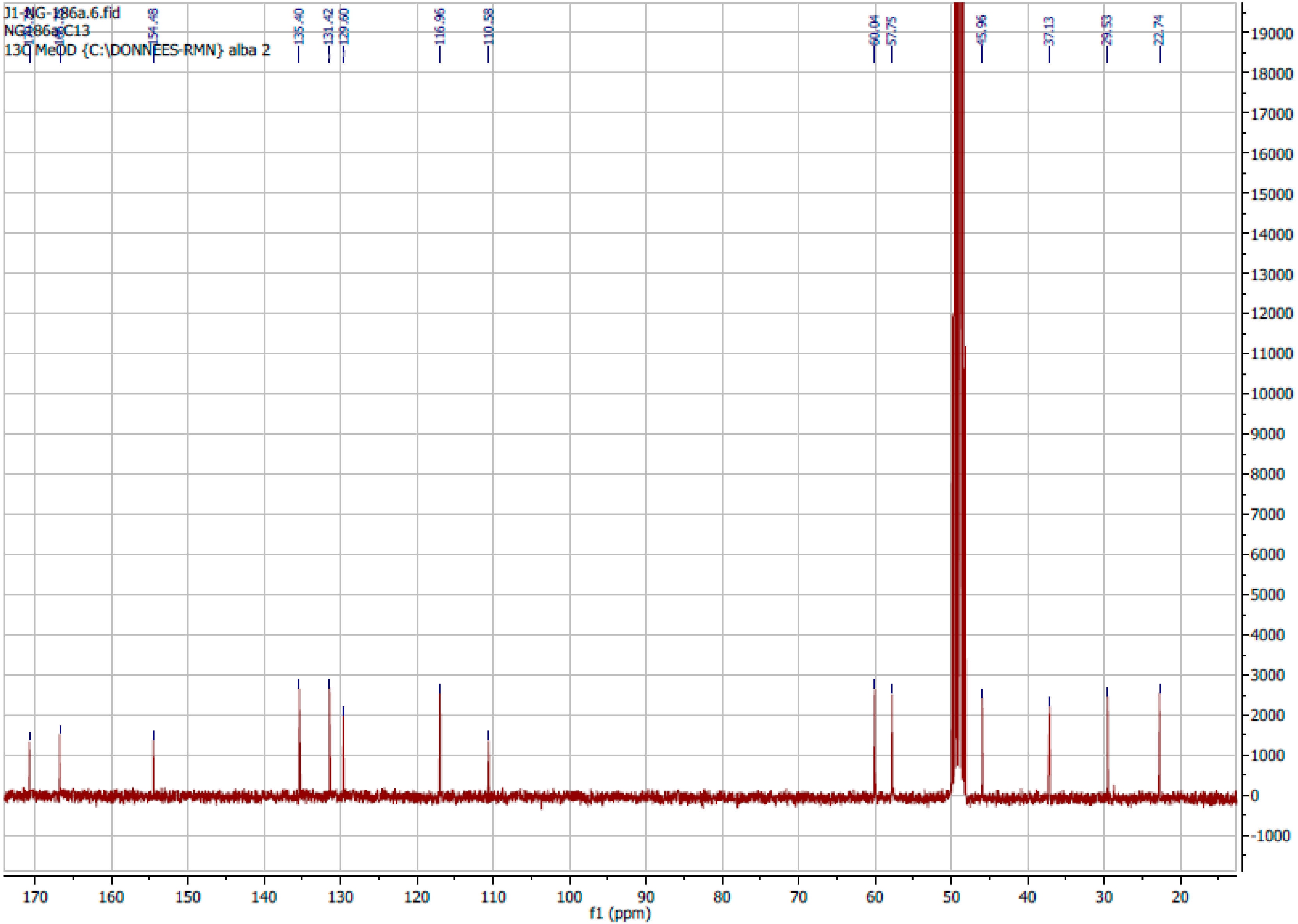

Figure S13: *^{13}C-NMR spectrum of compound* ***7*** *in CD_3OD.*

S14

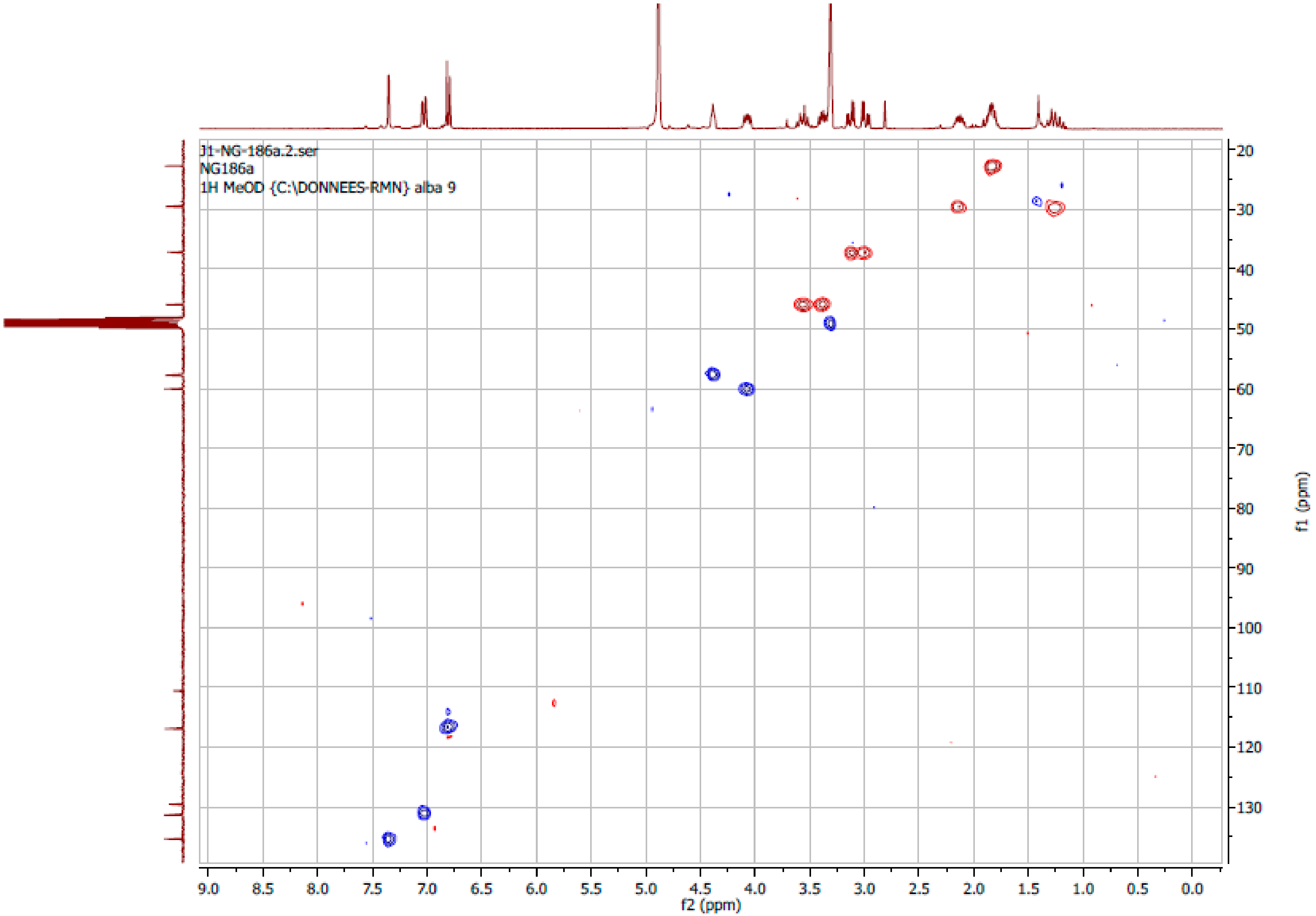

Figure S14: *HSQC spectrum of compound **7** in* CD_3OD.

S15

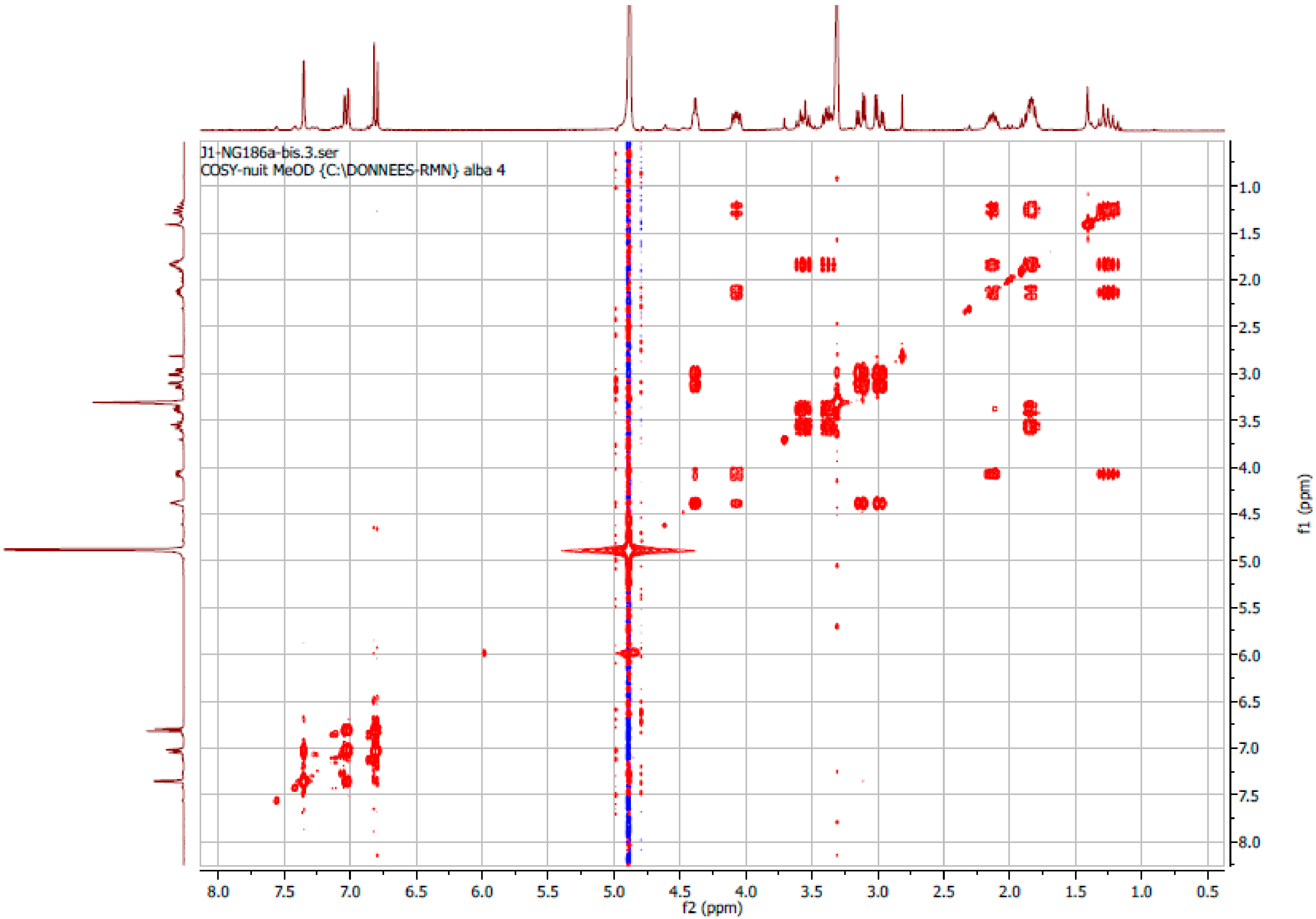

Figure S15: *COSY spectrum of compound **7** in CD_3OD.*

S16

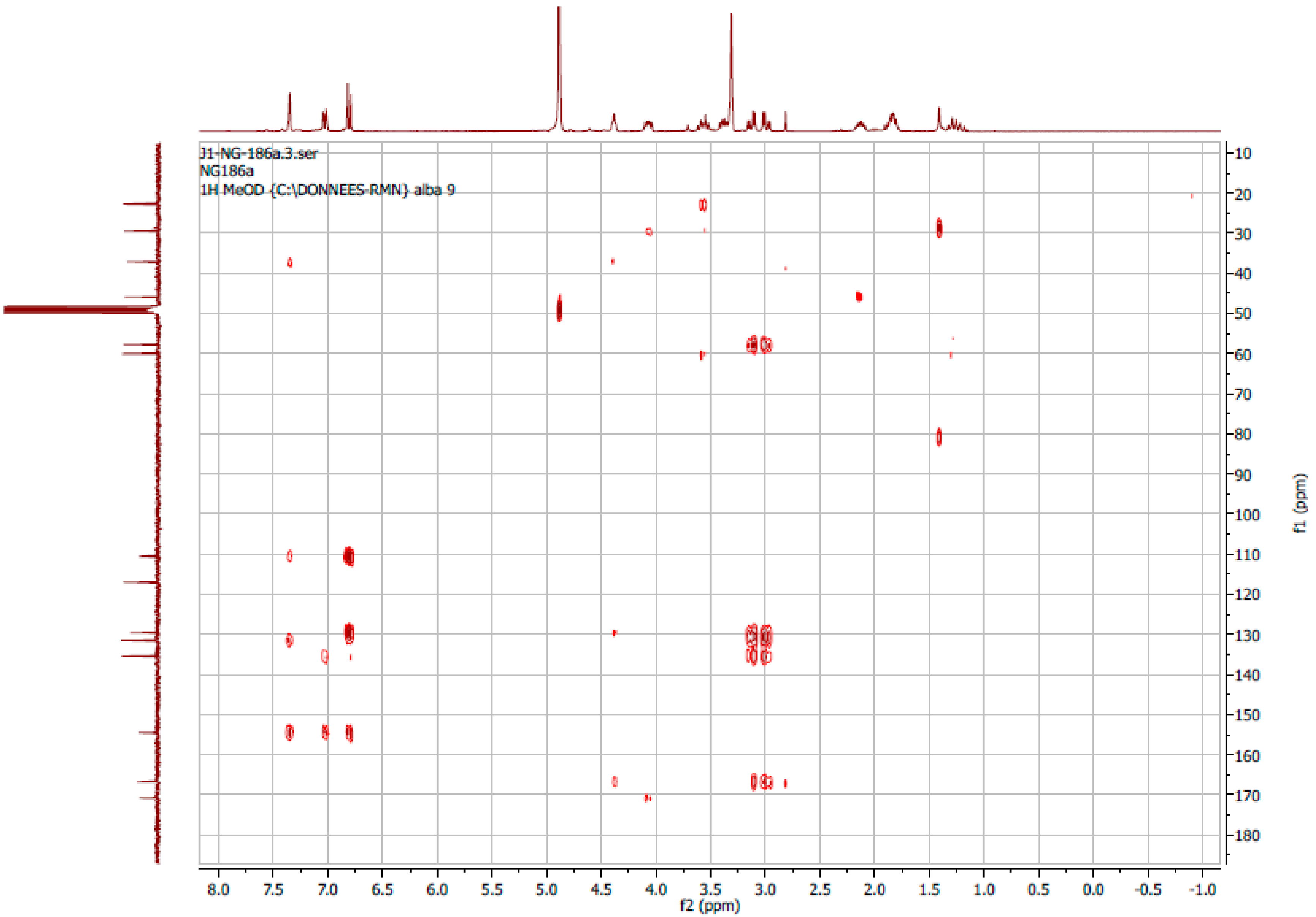

Figure S16: *HMBC spectrum of compound **7** in CD_3OD.*

S17

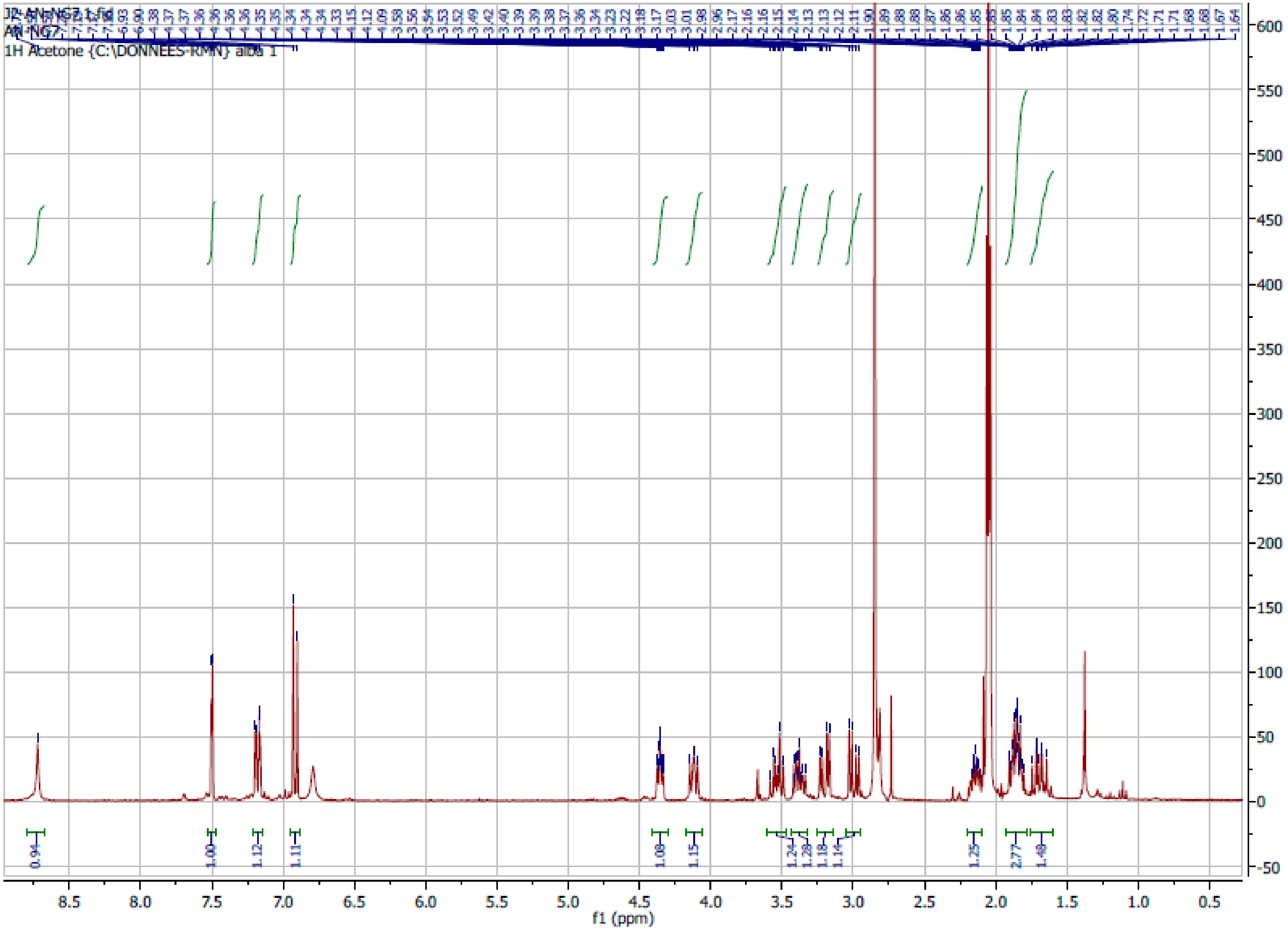

Figure S17: *^{1}H-NMR spectrum of compound **7** in acetone-d$_6$.*

S18

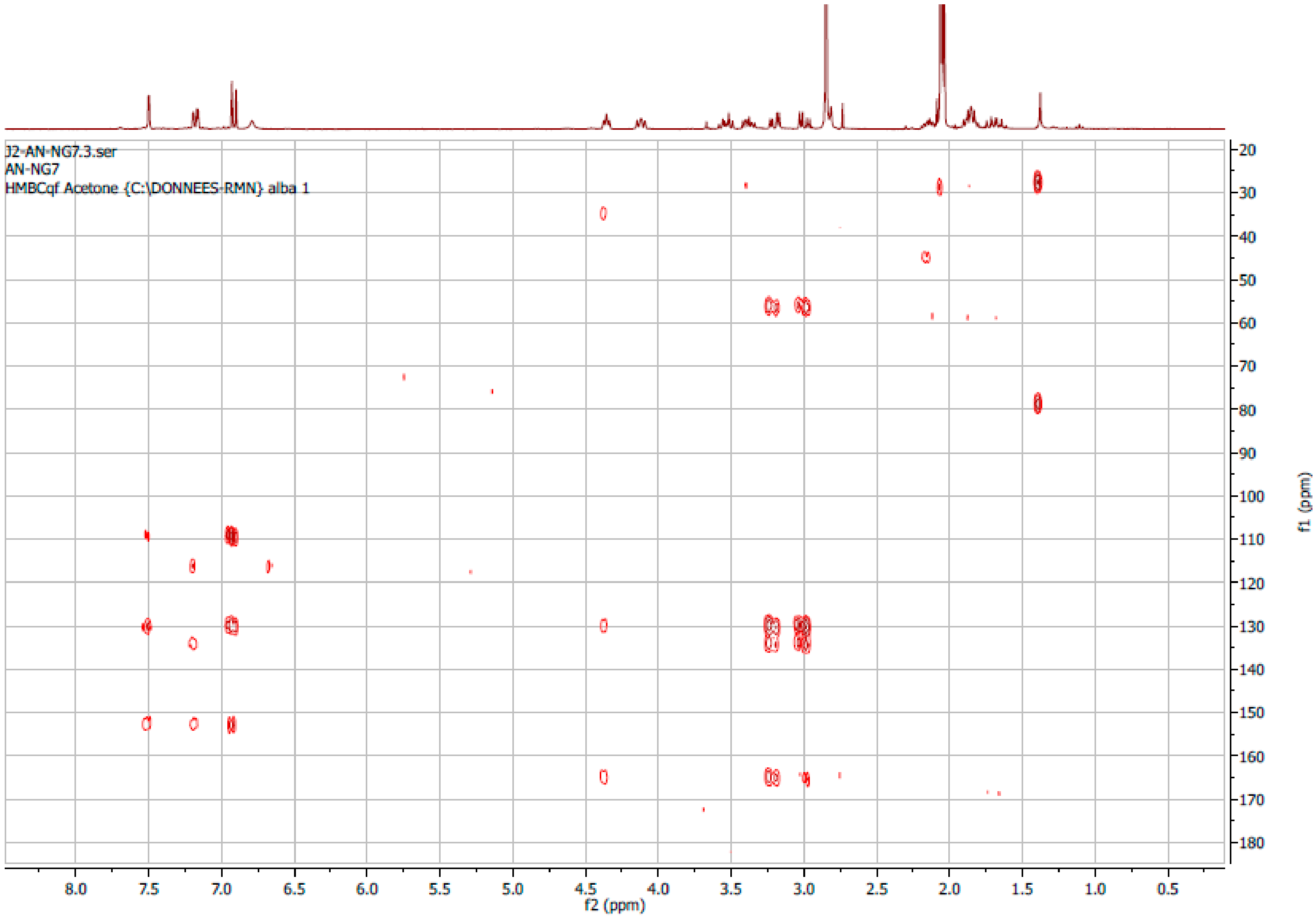

Figure S18 : *HMBC spectrum of compound* ***7*** *in acetone-d*$_6$.

S19

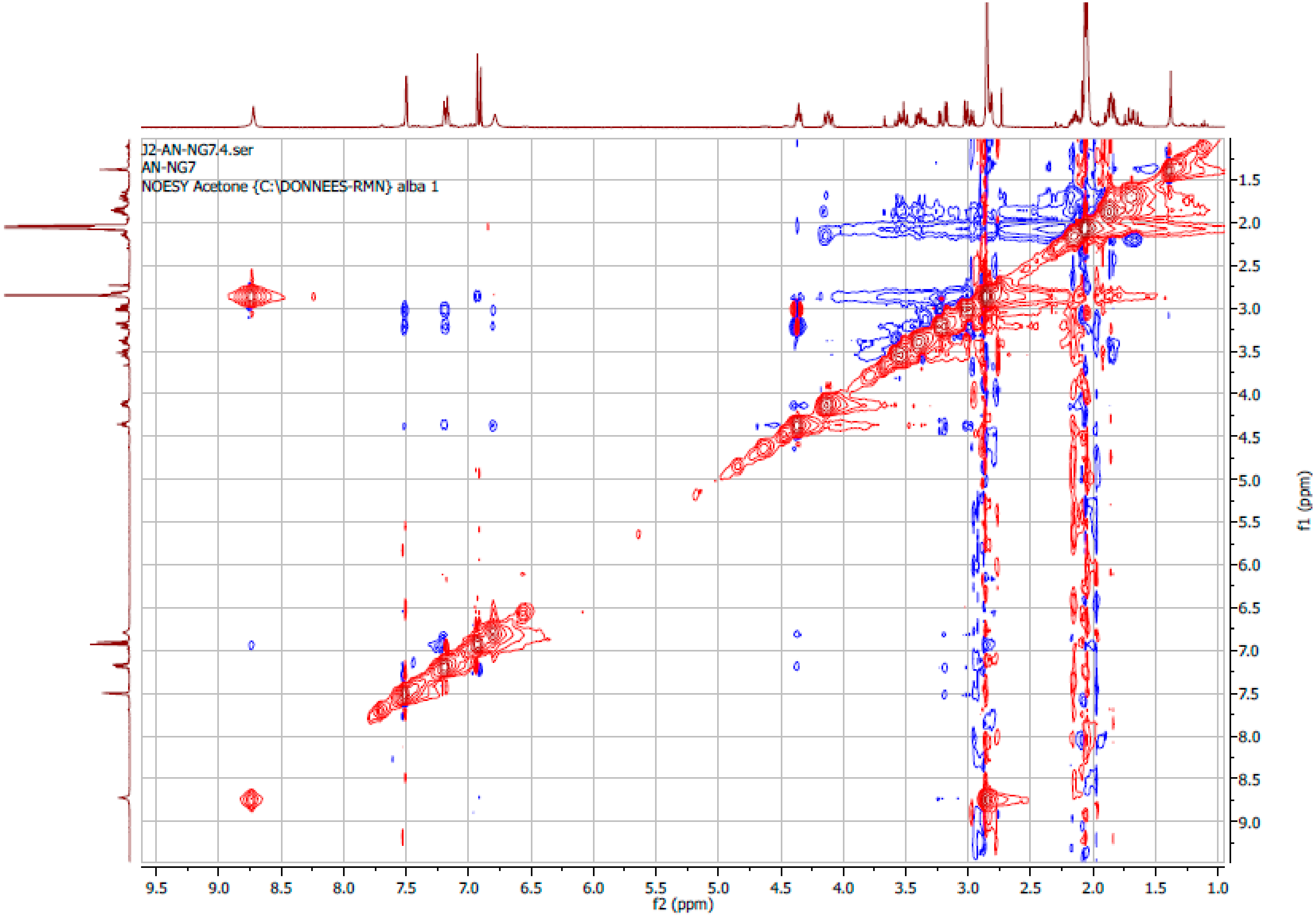

Figure S19: *NOESY spectrum of compound **7** in acetone-d₆.*

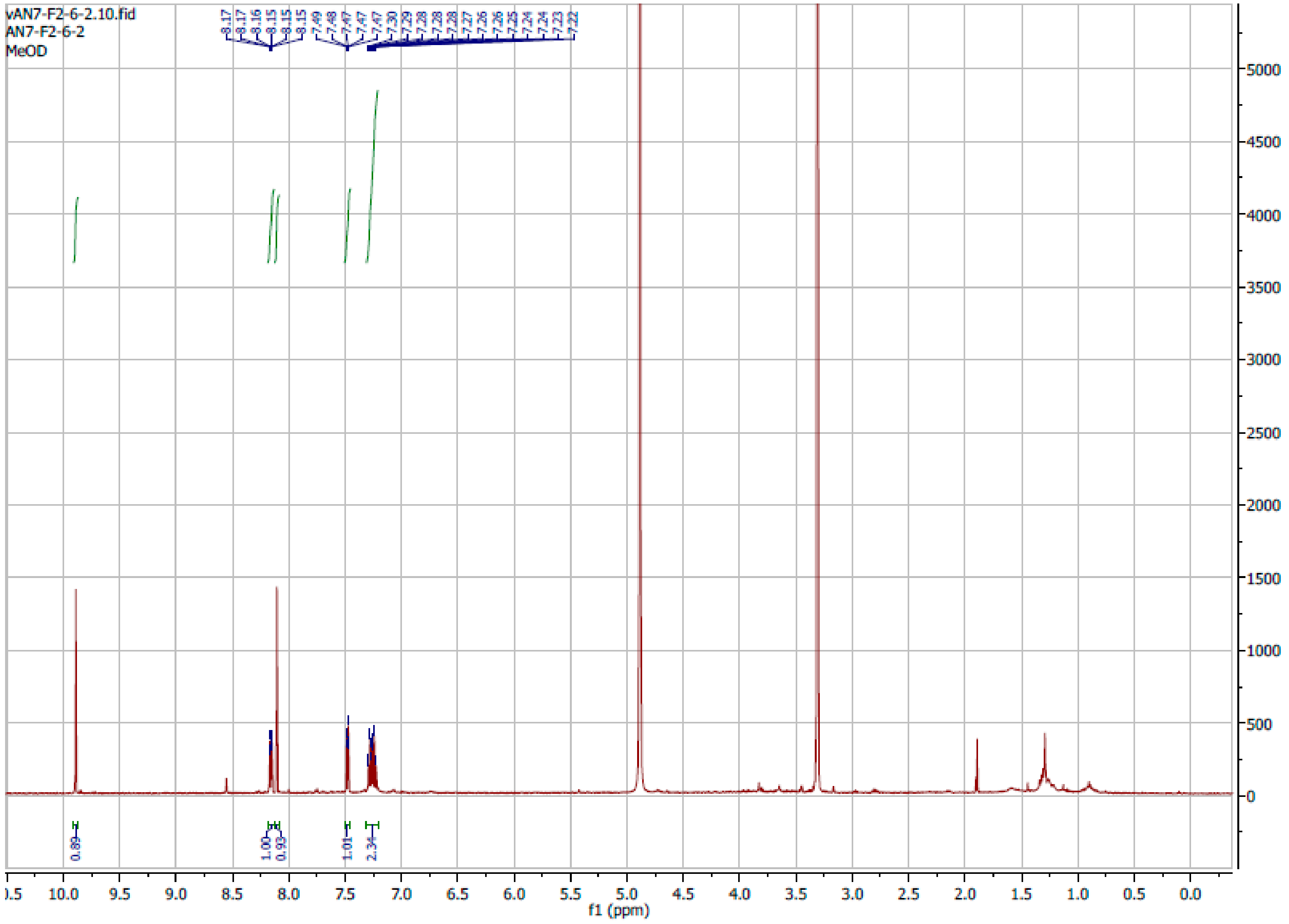

Figure S20: *^{1}H-NMR spectrum of compound **8** in CD_3OD.*

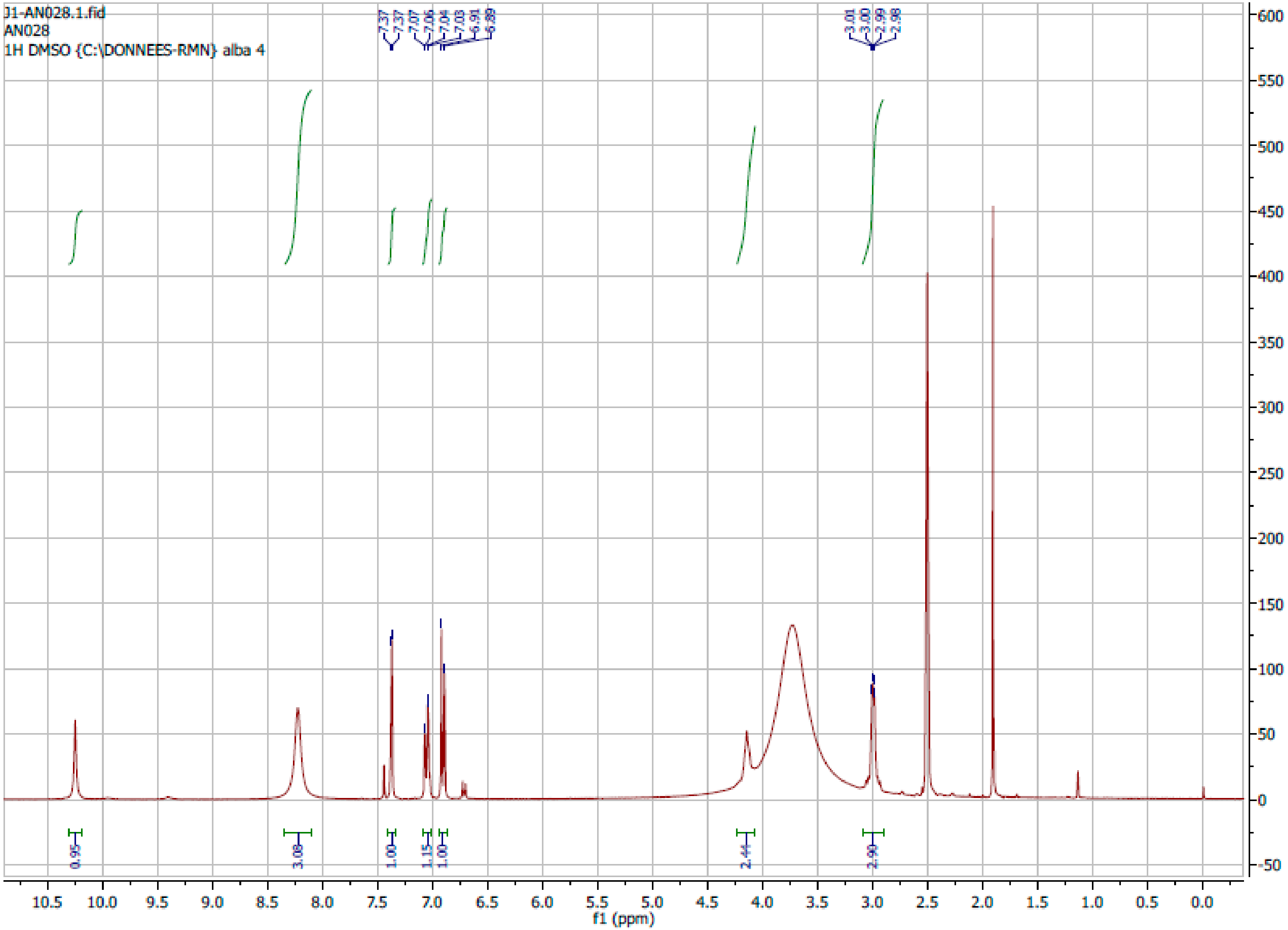

Figure S21: *^{1}H-NMR spectrum of compound* ***10*** *in DMSO-d*$_6$.

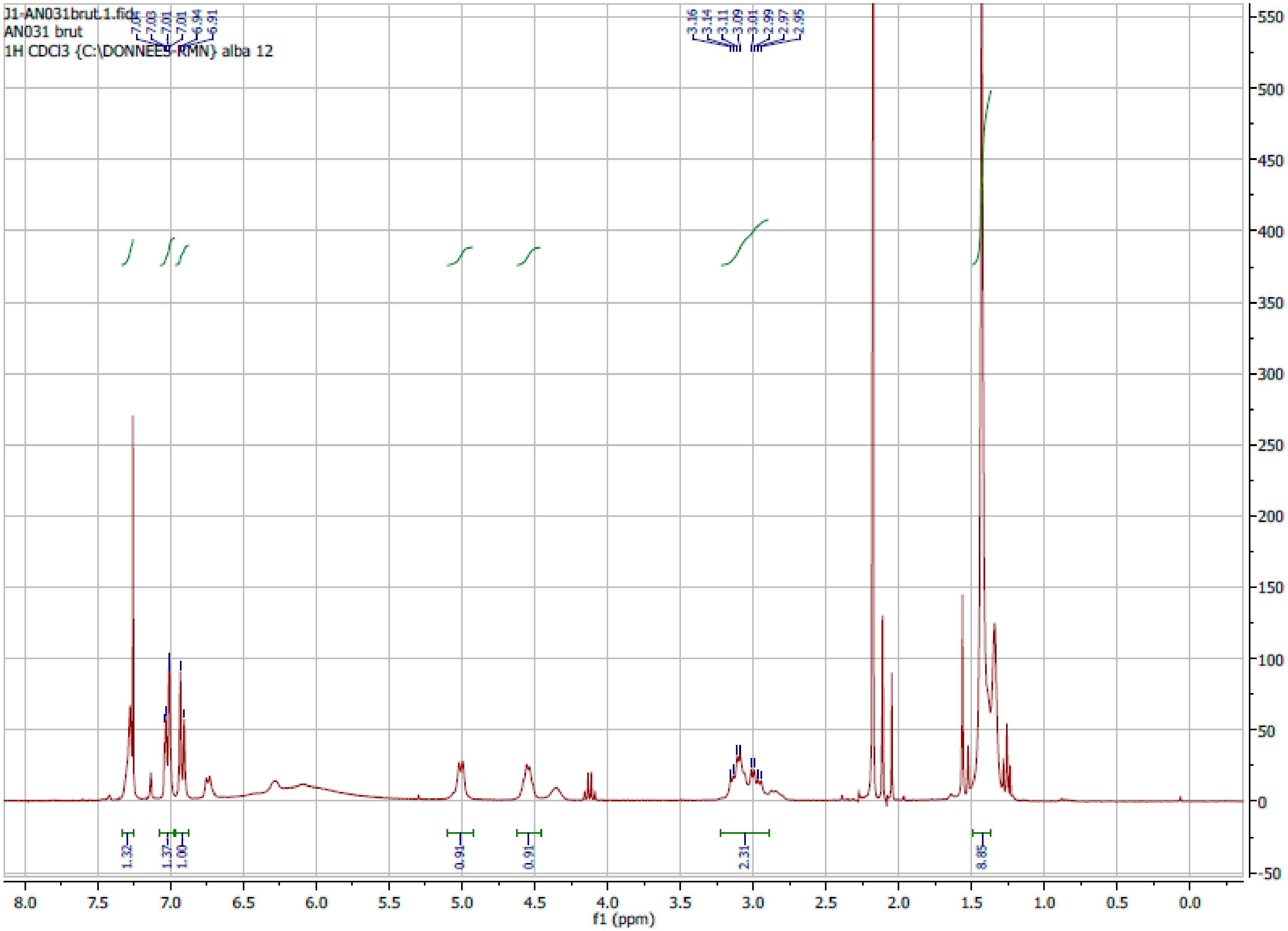

Figure S22: *^{1}H-NMR spectrum of compound* ***11*** *in CDCl₃.*

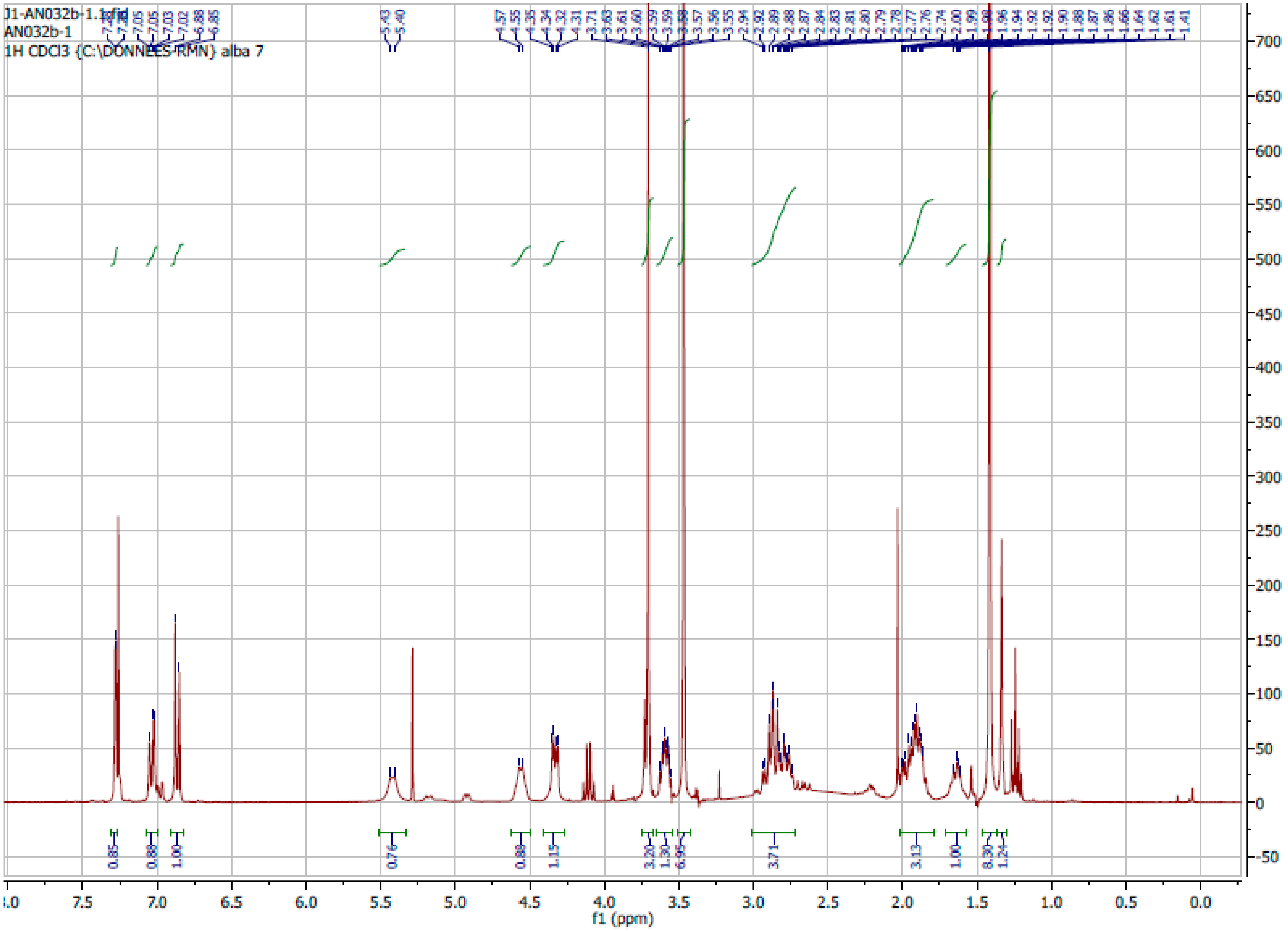

Figure S23: *¹H-NMR spectrum of compound* ***13a*** *in CDCl₃.*

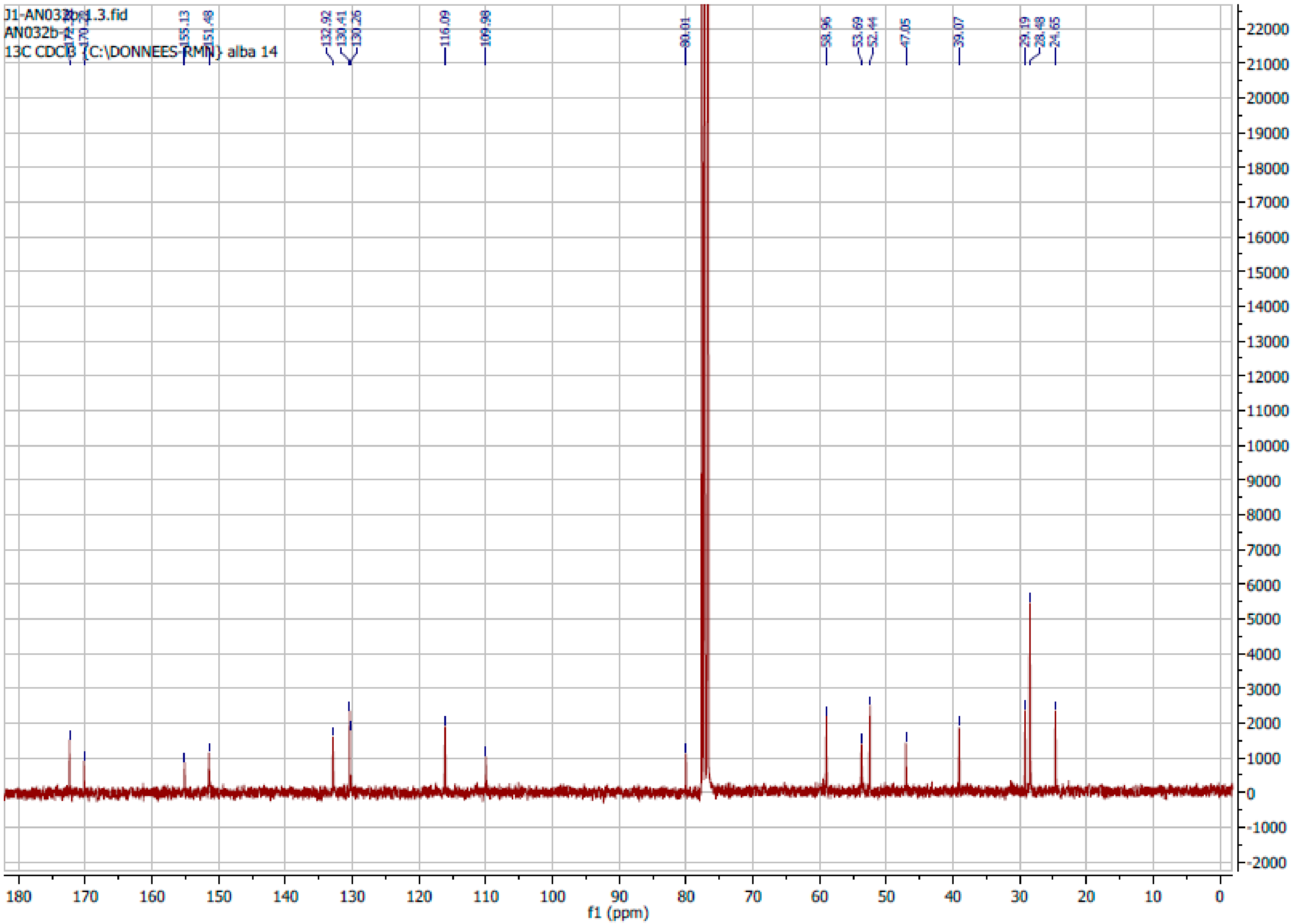

Figure S24: *^{13}C-NMR spectrum of compound **13a** in $CDCl_3$.*

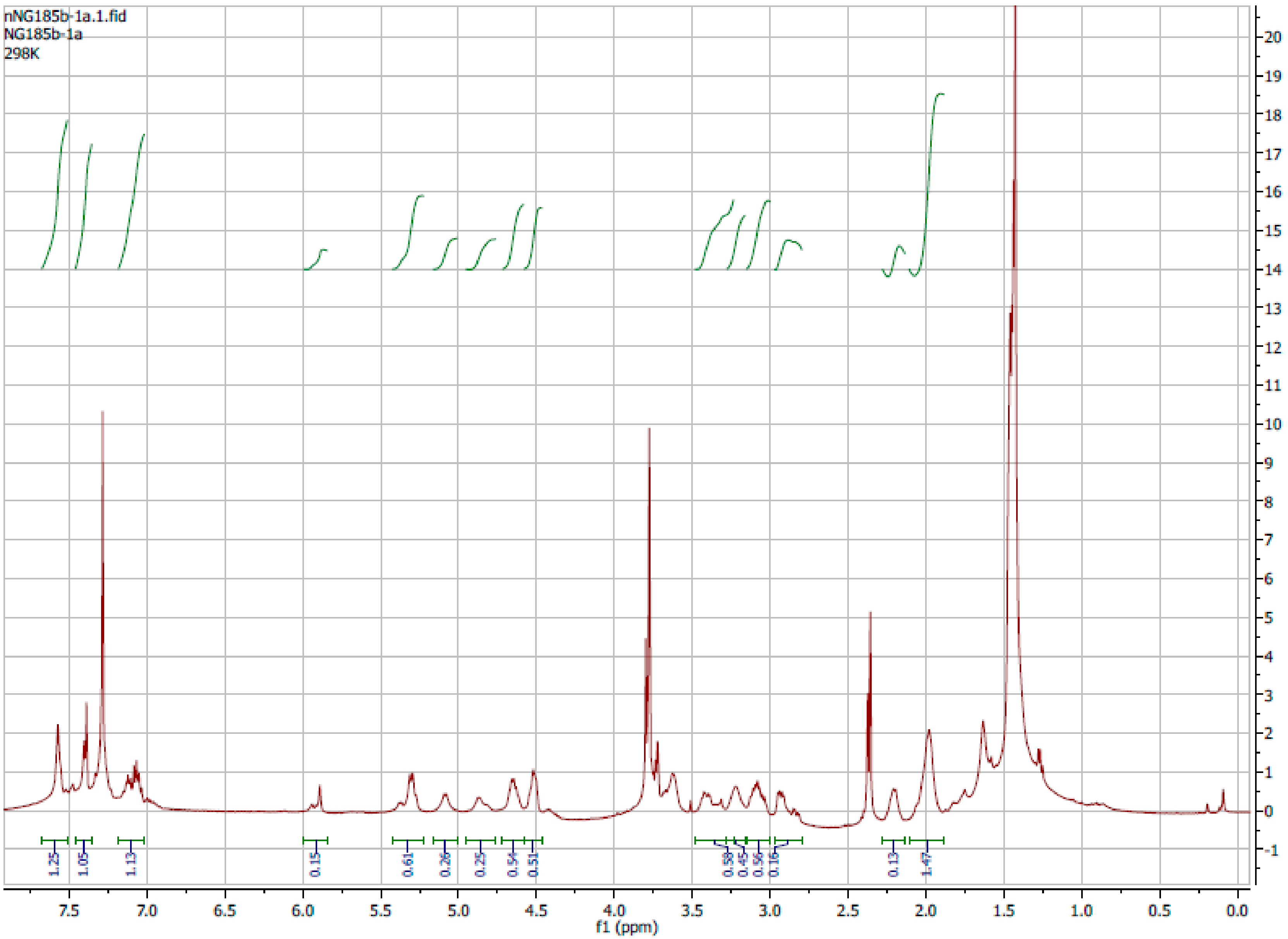

Figure S25: *^{1}H-NMR spectrum of compound* ***13b*** *in* $CDCl_3$*.*

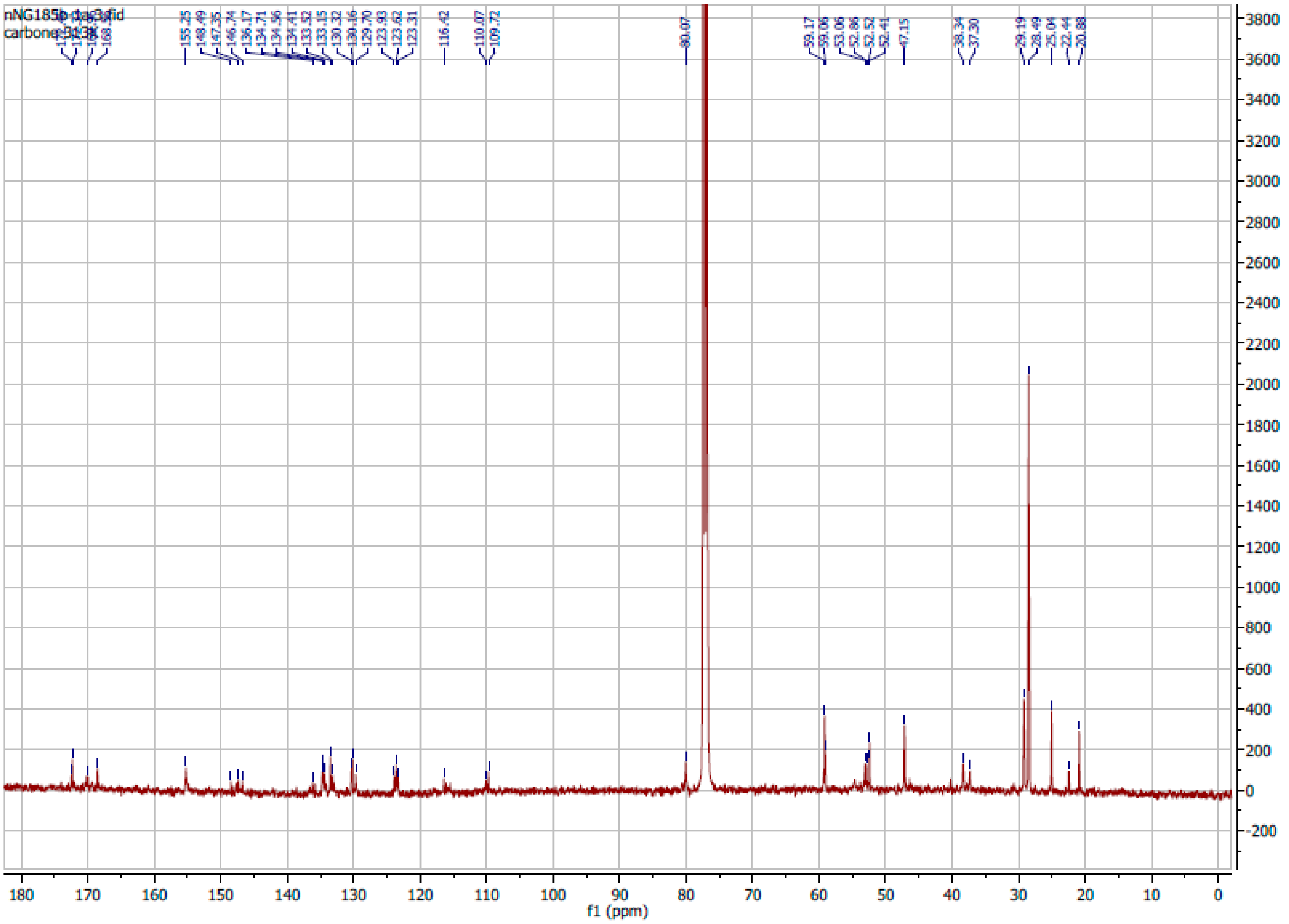

Figure S26: *^{13}C-NMR spectrum of compound* ***13b*** *in CDCl*$_3$*.*

S27

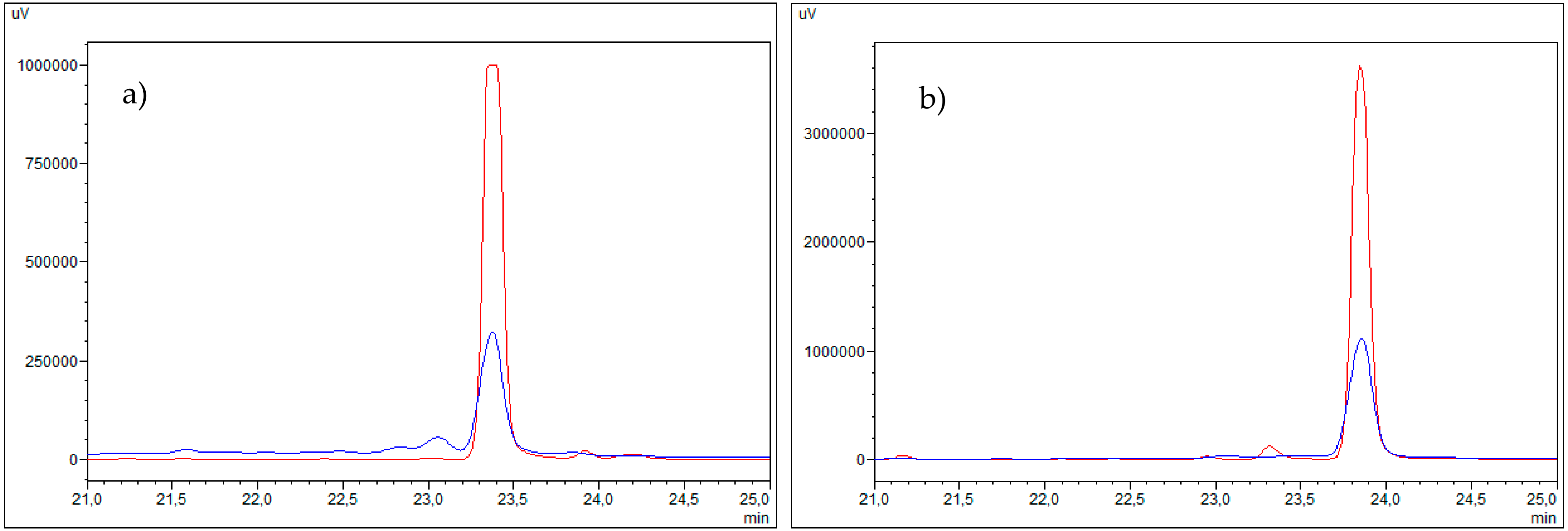

Figure S27: *HPLC chromatogram at 220 nm of isolated (blue) and synthetic (red) compounds* ***6*** *(**a**) and* ***7*** *(**b**).*
